# Cluster-Based Phenotyping in an HFpEF Population Identifies a Biomarker-High, Atrial-Dysfunction Phenotype with Impaired Quality of Life, Reduced Exercise Capacity and Higher Healthcare Utilization

**DOI:** 10.3390/jcm15197538

**Published:** 2026-09-28

**Authors:** Sophia Haack, Frank Edelmann, Fabian Spinka, Andreas Kind, Fabienne Beisegel, Leif-Hendrik Boldt, Abdul Shokor Parwani, Florian Blaschke, Felix Hohendanner, Gerhard Hindricks, Kevin Vernooy, Dominik Linz, Ingo Hilgendorf, Doreen Schoeppenthau

**Affiliations:** 1 Department of Cardiology, Angiology and Intensive Care Medicine, Deutsches Herzzentrum der Charité, Augustenburger Platz 1, 13353 Berlin, Germany; sophia.haack@charite.de (S.H.); frank.edelmann@dhzc-charite.de (F.E.);; 2 Charité-Universitätsmedizin Berlin, Freie Universität Berlin and Humboldt-Universität zu Berlin, Charitéplatz 1, 10117 Berlin, Germany; 3 DZHK (German Centre for Cardiovascular Research), Partner Site Berlin, 10117 Berlin, Germany; 4 Department of Cardiology, Angiology and Intensive Care Medicine, Deutsches Herzzentrum der Charité, Charitéplatz 1, 10117 Berlin, Germany; 5 Department of Physiology, Cardiovascular Research Institute Maastricht (CARIM), Maastricht University, 6229 ER Maastricht, The Netherlands; 6 Department of Cardiology, Cardiovascular Research Institute Maastricht (CARIM), Maastricht University Medical Centre (MUMC+), 6229 HX Maastricht, The Netherlands; 7 Department of Biomedical Sciences, Faculty of Health and Medical Sciences, University of Copenhagen, DK-2200 Copenhagen, Denmark; 8 Berlin Institute for the Foundations of Learning and Data (BIFOLD), Technical University of Berlin, 10587 Berlin, Germany

**Keywords:** heart failure with preserved ejection fraction, atrial cardiomyopathy, left atrial strain, left atrial stiffness, atrial fibrillation, phenotyping, cluster analysis

## Abstract

**Background/Objectives:** Heart failure with preserved ejection fraction (HFpEF) is a heterogeneous syndrome. Atrial remodeling represents an important dimension of HFpEF beyond the presence of atrial fibrillation (AF). We aimed to identify clinically meaningful atrial phenotypes using unsupervised clustering. **Methods:** Overall, 153 patients were analyzed from a prospective HFpEF registry who underwent clinical assessment, laboratory testing, electrocardiography, resting and stress echocardiography and cardiopulmonary exercise testing. Consensus k-means clustering across multiply imputed datasets integrated markers of atrial structure and function, biomarkers, and hemodynamic burden. Clinical characteristics, functional capacity, quality of life and 12-month healthcare utilization were compared between clusters. Sensitivity analyses assessed cluster robustness. **Results:** Two phenotypes were identified. Cluster 1 (n = 66) was characterized by higher biomarker and hemodynamic burden, impaired renal function, larger left atrial volume index, and reduced left atrial reservoir strain. Variables not included in cluster generation also differed between phenotypes: Cluster 1 showed poorer exercise capacity, shorter six-minute walking distance, worse quality of life, and a higher observed all-cause hospitalization burden. The hospitalization finding was considered exploratory. The principal cluster structure was preserved in sensitivity analyses, including complete-case analysis. **Conclusions:** The clustering approach identified an HFpEF phenotype characterized by biomarker elevation and advanced atrial remodeling. The phenotype was associated with greater functional impairment and adverse patient-reported characteristics. These clinical associations support the potential relevance of multidimensional atrial phenotyping, although the findings remain hypothesis-generating and require validation in independent cohorts.

## 1. Introduction

Heart failure with preserved ejection fraction accounts for approximately half of all cases of heart failure [1,2]. Patients with HFpEF have a poorer prognosis, higher rehospitalisation rates and a substantial burden of comorbidities [2]. Furthermore, HFpEF is characterized by clinical and pathophysiological heterogeneity, making it a challenging cardiovascular syndrome [3]. Patients often live with reduced physical capacity and impaired quality of life, despite a preserved left ventricular ejection fraction [4].

In recent years, increasing attention has been paid to the left atrium (LA), as it can reflect the effects of diastolic function [5]. Through its reservoir, conduction and booster pump function, the LA reflects left ventricular relaxation, filling pressures and cardiovascular load [6]. Atrial remodeling, driven by structural, electrical and functional changes, is hypothesized to correlate with the severity and progression of HFpEF [7].

Also, AF is common in HFpEF, illustrating how complex and close the relationship between these two conditions is [8,9]. It may arise as a result of atrial remodeling and, at the same time, accelerate the structural and functional deterioration of the atria, creating a bidirectional feedback loop that contributes to haemodynamic instability [10]. However, patients with HFpEF are also characterized by a high prevalence of obesity, high blood pressure, renal dysfunction, diabetes and other comorbidities, which promote myocardial and atrial remodeling regardless of heart rhythm [11]. Although AF is easily detectable, new evidence suggests that subclinical atrial dysfunction is also common in patients with HFpEF who remain in sinus rhythm (SR) [12]. It therefore remains unclear whether atrial dysfunction in HFpEF is primarily attributable to AF or whether it represents a broader structural and systemic disease phenotype. The exact relationships between electrical changes, mechanical impairments (such as reduced left atrial reservoir strain (LASr) or an impaired left atrial ejection fraction (LAEF)), atrial enlargement and biochemical stress markers have not yet been fully clarified. Furthermore, stress echocardiography may provide additional information on exercise-related changes in atrial function beyond resting measurements [13].

To address these questions, the present study employs a multimodal phenotyping approach that integrates echocardiographic measurements of the structure and function of the LA, exercise parameters and clinical laboratory parameters in a well-characterized HFpEF cohort. Using cluster modeling, we sought to identify different profiles of atrial remodeling and to characterize their clinical relevance. Our aim was to determine whether atrial dysfunction is primarily associated with the presence of AF or forms part of a broader structural phenotype linked to the burden of comorbidity, hemodynamic stress and impaired functional capacity. In addition, stress echocardiography was used to descriptively characterize changes in left atrial function during exercise. A more comprehensive understanding of the multidimensional nature of atrial remodeling in HFpEF could provide deeper insights into the mechanisms underlying disease heterogeneity and support more precise clinical characterization and future hypothesis-driven studies.

## 2. Methods

### 2.1. Study Population

For this study, we used the HFpEF registry of Charité University Medicine at Virchow Hospital in Berlin. A total of 153 patients were recruited between November 2016 and September 2021 based on the following inclusion and exclusion criteria:

Participants were eligible for inclusion if they were ≥ 18 years of age and provided written informed consent. Patients with preserved left ventricular ejection fraction (LVEF ≥ 40%), New York Heart Association (NYHA) functional class ≥ II, and NT-proBNP levels ≥ 125 pg/mL were included. If echocardiographic data were older than three months, reassessment was performed.

In addition, at least one of the following echocardiographic criteria had to be present: E/e′ ≥ 13 in combination with a reduced e′ velocity (<9 cm/s, mean of septal and lateral measurements), LAVI > 34 mL/m^2^, or increased left ventricular mass index (LVMI ≥ 115 g/m^2^ in men or ≥ 95 g/m^2^ in women).

Patients were excluded if they were unable or unwilling to provide informed consent, had end-stage renal disease requiring dialysis, or had experienced a myocardial infarction (ST-elevation myocardial infarction (STEMI) or non-ST-elevation myocardial infarction (NSTEMI)) or underwent cardiac surgery/intervention within the previous three months. Further exclusion criteria included hemodynamically relevant pericardial disease and inability to complete study visits during the one-year follow-up period. For this study, we used data from patients’ initial consultations and follow-up telephone consultations one year later.

At the time of inclusion, the criterion LVEF ≥ 40% was used. This includes patients with LVEF between 40% and 49%, who would not meet the contemporary definition of HFpEF [14]. Because of that, an additional sensitivity analysis was performed in patients with LVEF ≥ 50%.

### 2.2. Variables

Baseline patient characteristics were collected during the initial study visit using standardized questionnaires, laboratory testing, electrocardiography (ECG), transthoracic echocardiography, stress echocardiography, and cardiopulmonary exercise testing (CPET). Follow-up data regarding hospitalizations, medical contacts within 12 months and questionnaires were obtained during a structured telephone interview. Hospitalizations were not prospectively adjudicated by a physician. All-cause hospitalization was therefore used as the principal hospitalization outcome. For categorical analyses, MLHFQ total scores were classified as good (<24 points), moderate (24–45 points), or poor (>45 points) health-related quality of life according to previously proposed cut-off values [15].

#### 2.2.1. Electrocardiography Parameters

All ECGs recorded at the baseline visit were analyzed, and recordings in SR were identified for the measurement of P-wave parameters. Standard ECG parameters (heart rate, QRS duration, P electrical axis, QT duration) were taken from automated machine readouts. P-wave parameters were measured manually by two independent observers. The following parameters were selected: P-wave duration (lead II and V1), P-wave amplitude, P-terminal force in V1 (PTFV1), P-wave dispersion and interatrial block. Definitions and reference thresholds for P-wave parameters were based on the consensus recommendations by Chen et al [16].

#### 2.2.2. Echocardiography Parameters

Echocardiographic examinations were performed by an experienced echocardiographer using standard clinical equipment (Vivid E95, Vivid7, GE Healthcare, Chicago, IL, USA). Echocardiography was performed in accordance with the recommendations of American Society of Echocardiography [17]. Echocardiographic image acquisition followed a standardized protocol with optimization of image quality and digital storage for offline analysis. In patients in sinus rhythm, at least three complete cardiac cycles were recorded, and two-dimensional images were acquired at a frame rate of 50–80 frames/s. Analyses were performed using dedicated post-processing software (TomTec Imaging Systems, Version TTA2.51.04, Unterschleißheim, Germany). Speckle-tracking contours were visually inspected, and manual adjustments of the endocardial border were performed when necessary to ensure adequate tracking. Left atrial strain analysis was performed using the R-R interval as the temporal reference, based on the method described by Javadi et al. [18]. In patients with atrial fibrillation at the time of echocardiographic acquisition, two measurements were obtained and averaged to account for beat-to-beat variability. Measurements with inadequate tracking despite manual correction were considered non-evaluable. Left atrial stiffness was calculated as the ratio of mean E/e′ to the absolute value of four-chamber left atrial reservoir strain (LASr): $LA\;Stiffness\;index=mean\;E/e^{\prime }÷\;\left|LASr\right|$ [19].

#### 2.2.3. Cardiopulmonary Exercise Testing

Cardiopulmonary exercise testing (CPET) was performed on an electronically cycle ergometer using a stepwise protocol starting at 10 W, with workload increased by 10 W every minute while a cadence of approximately 60 rpm (acceptable range 55–65 rpm) was maintained. Gas-exchange variables, including oxygen uptake (VO_2_), carbon dioxide production (VCO_2_), minute ventilation (VE), and the respiratory exchange ratio (RER), were continuously recorded and processed as 10 s averages. Twelve-lead ECG was continuously monitored, and blood pressure and perceived exertion using the Borg scale were assessed during exercise. Exercise was continued until symptom limitation, or predefined clinical termination criteria were reached. The protocol specified an RER >1.0 at the end of exercise as an effort criterion; when the resting RER was already >1.0, an increase of 0.3 from baseline was considered acceptable. Peak VO_2_ was recorded as absolute oxygen uptake in l/min. Maximal workload, maximal RER, VE/VCO_2_ slope, Borg score, and the reason for test termination were documented.

### 2.3. Clustering Approach

The following variables were included in the clustering analysis: clinical characteristics (BMI), echocardiographic parameters (LAVI (biplane), LASr in the 4-chamber view, mean E/e′, LVMI, Tricuspid regurgitation-gradient (TR-gradient)), laboratory parameters (NT-proBNP, estimated glomerular filtration rate (eGFR), glycated hemoglobin (HbA1c), and hemoglobin). All variables included in the clustering procedure were assessed at baseline prior to outcome evaluation.

Missing data in the clustering variables were handled using multiple imputations by chained equations [20,21]. The imputation model included the same ten variables used for phenotype clustering. Missing continuous values were imputed using predictive mean matching. Twenty imputed datasets were generated with 20 iterations per imputation. For each incomplete clustering variable, the remaining clustering variables were used as predictors in the imputation model. Clinical outcome variables were not included in the imputation model. Multiple imputation was performed under the assumption that missingness was conditionally missing at random given the observed variables included in the imputation model.

K-means clustering was then performed independently in each imputed dataset, and the resulting partitions were combined into a patient-level consensus assignment following the general framework for clustering multiply imputed datasets described by Audigier and Niang [22] (Figure 1). Because cluster labels are arbitrary across independently fitted k-means solutions, labels were aligned to a reference partition before final cluster membership was determined by majority voting across all imputed datasets. As a sensitivity analysis, the clustering procedure was repeated in patients with complete data for all ten clustering variables.

Before clustering, all continuous variables were standardized using z-score transformation to ensure comparability across different measurement scales. K-means was selected because of its robustness, reproducibility, and clinical interpretability in multidimensional datasets, and because it has previously been applied for phenotype identification in cardiovascular research [23]. Cluster stability was assessed by calculating the ARI between each imputation-specific partition and the final consensus solution. Solutions with k = 2–6 were evaluated using PAC, mean silhouette width, and bootstrap-based Jaccard stability. Cluster instability was defined as 1 − ARI, and patient-level consensus strength as the proportion of imputed datasets in which the aligned cluster assignment agreed with the final consensus assignment.

### 2.4. Statistical Analysis

Baseline characteristics were compared between clusters using descriptive statistical methods. Continuous variables are presented as mean ± standard deviation (SD) or median (interquartile range (IQR)), as appropriate. Continuous variables were compared using Welch’s t-test. Individual MLHFQ items were analyzed using the Wilcoxon rank-sum test because of their ordinal nature. Categorical variables were shown as frequencies and percentages and compared using the chi-square test or Fisher’s exact test, as appropriate. Effect sizes are reported as Hedges’ g for continuous comparisons, rank-biserial correlations for Wilcoxon comparisons, and Cramér’s V for categorical comparisons. Effect sizes were interpreted in terms of magnitude, with larger absolute values indicating greater between-cluster differences; the direction of the sign reflects the direction of the comparison (Cluster 1 minus Cluster 2).

Ordinal logistic regression was used for ordered categorical outcomes, including quality-of-life categories and follow-up NYHA class. The latter model was adjusted for baseline NYHA class. Binary logistic regression was used for dichotomous clinical outcomes, including all-cause hospitalization, general practitioner as well as cardiologist visits. All-cause hospitalization was dichotomized as ≥1 versus no hospitalization and compared between clusters. General practitioner and cardiologist visits were categorized as ≥ 2 versus 0–1 visit. Sensitivity analyses were performed by excluding individual clustering variables, including LAVI and NT-proBNP, to assess the robustness of the identified phenotype. In an additional sensitivity analysis, comparisons were repeated in patients who were in sinus rhythm during resting echocardiography and for patients with no missing values in the variables for the cluster. For the LVEF ≥ 50% sensitivity analysis the complete multiple-imputation and clustering procedure was repeated using the same ten variables and identical analytical settings as in the primary analysis. Cluster labels were aligned with those of the primary analysis, and concordance was quantified using the adjusted Rand index (ARI). Clinical outcomes were reevaluated within the restricted cohort.

All statistical tests were two-sided, and a *p*-value < 0.05 was considered statistically significant. For the 21 individual MLHFQ items, false discovery rate control was performed using the Benjamini-Hochberg procedure; raw *p*-values, BH-adjusted q-values, and rank-biserial effect sizes are reported in Supplementary Table S3. Effect sizes were additionally analyzed. Statistical analyses were performed using R (R Foundation for Statistical Computing, Vienna, Austria; version 2026.4.0.526).

## 3. Results

### 3.1. Identification and Cluster-Defining Characteristics of the Consensus Clusters

After statistical analysis a two-cluster solution was selected. Cluster 1 included 66 patients (43.1%), whilst Cluster 2 comprised 87 patients (56.9%). Of the 153 patients, 107 had complete data for all clustering variables, whereas at least one value was missing for 46 patients (30.1%). Variable-specific missingness is reported in Supplementary Table S4.

The cluster profile showed that Cluster 1 was characterized by higher NT-proBNP levels, a higher LAVI (Figure 2A), increased E/e′ and TR pressure gradients, as well as a lower eGFR and reduced LASr. In comparison, BMI, LVMI and hemoglobin had a smaller influence on cluster differentiation (Figure 2A). Cluster 1 was interpreted as a phenotype characterized by high biomarker levels (Figure 2B), cardiorenal dysfunction and atrial dysfunction, while Cluster 2 represented a phenotype with a lower haemodynamic and biomarker burden and better-preserved atrial function (Table 1). Because these variables were used to generate the clustering solution, their between-cluster differences represent defining characteristics of the identified phenotypes and were not considered independent validation of the cluster solution.

### 3.2. Cluster Stability and Internal Reproducibility

The final two-cluster model showed high reproducibility, with a mean ARI of 0.91 and a median ARI of 0.95. Median patient-level consensus strength was 1.00, indicating highly consistent cluster assignments across imputations. Consensus diagnoses across different solutions with k = 2–6 further supported the choice of a two-cluster solution, which exhibited the highest bootstrap cluster stability, the lowest proportion of ambiguous clustering and the highest mean silhouette width (Supplementary Figure S1). A two-cluster solution was therefore selected based on its clinical interpretability and robust reproducibility and is illustrated in the principal component analysis (PCA) plot (Figure 3). PCA was performed for visualization of the multidimensional cluster structure and did not contribute to cluster assignment. The clustering variables were used to describe the defining phenotype profiles, whereas variables not included in cluster generation were examined separately to assess the clinical characteristics associated with cluster membership.

### 3.3. Sensitivity Analyses

#### 3.3.1. Exclusion of LAVI

To assess whether the identified phenotype was predominantly driven by left atrial enlargement, a sensitivity analysis was performed excluding LAVI from the clustering model. The two-cluster structure remained similar, although cluster stability was moderately reduced compared with the full model (mean ARI 0.829 vs. 0.908; instability 0.171 vs. 0.092). Cluster sizes changed from 66 and 87 patients in the full model to 81 and 72 patients in the model without LAVI. 138 of 153 patients (90.2%) retained their original cluster assignment, while 15 patients (9.8%) shifted from Cluster 2 to Cluster 1. Overall, agreement between the two clustering solutions was substantial (ARI 0.644, Cohen’s κ = 0.805).

After excluding LAVI from the clustering model, LAVI remained significantly higher in Cluster 1. Patients in Cluster 1 displayed a higher mean LAVI than patients in Cluster 2 (49.7 ± 22.5 vs. 39.7 ± 9.5 mL/m^2^; *p* < 0.001) (Figure 4A). The standardized mean difference for LAVI between clusters was 0.57, indicating a moderate effect size. These findings indicate that LAVI contributes to cluster structure but that the two-cluster solution was not solely determined by left atrial enlargement.

#### 3.3.2. Exclusion of NT-proBNP

Exclusion of NT-proBNP substantially reduced the stability of the two-cluster solution. The mean ARI decreased from 0.908 in the full model to 0.562, while instability increased from 0.092 to 0.438. Cluster sizes changed from 66 and 87 patients in the full model to 72 and 81 patients in the model without NT-proBNP. After alignment of cluster labels, 125 of 153 patients (81.7%) retained their original cluster assignment, whereas 28 patients (18.3%) were reassigned. Of the patients initially assigned to Cluster 1, 55 of 66 (83.3%) remained in Cluster 1, while 70 of 87 patients (80.5%) initially assigned to Cluster 2 retained their assignment. These findings indicate that NT-proBNP substantially contributes to cluster stability and discrimination, although the underlying two-cluster structure persists in its absence.

#### 3.3.3. Complete-Case Analysis

A complete-case sensitivity analysis was performed in the 107 patients (69.9%) with complete data for all ten clustering variables. De novo k-means clustering again identified two groups comprising 59 and 48 patients. After alignment of cluster labels, 94 of 107 patients (87.9%) retained their original cluster assignment, corresponding to a moderate ARI of 0.569. The phenotypic direction of the cluster profiles was preserved, with Complete-case Cluster 1 showing higher LAVI, mean E/e′, TR pressure gradient, and NT-proBNP, together with lower LA reservoir strain and eGFR. Thus, although the two partitions were not identical, the principal cluster structure was reproduced in the absence of imputation (Supplementary Table S5).

#### 3.3.4. Sensitivity Analysis in Patients with LVEF ≥ 50%

LVEF was available in 152 of 153 patients; 138 (90.8%) patients had LVEF ≥ 50%, but 14 (9.2%) had LVEF between 40% and 49%. Repeating the clustering procedure in the LVEF ≥ 50% subgroup returned two clusters including 56 and 82 patients. Cluster assignment was highly concordant with the primary analysis: 135 of 138 patients (97.8%) preserved their original cluster, corresponding to an adjusted Rand index of 0.914. Between-cluster differences in functional capacity and health-related quality of life were also preserved in this cohort. In contrast all-cause hospitalization did not reach statistical significance (OR 1.75, 95% CI 0.61–5.13; *p* = 0.331; Supplementary Table S7).

### 3.4. Baseline Clincial Characteristics and Comorbidities

Patients in Cluster 1 were significantly older than those in Cluster 2 (75.98 ± 5.69 vs. 70.98 ± 8.23 years; *p* < 0.001). No significant differences were observed in terms of sex distribution or body mass index.

A history of AF was more common in Cluster 1 than in Cluster 2 (72.7% vs. 50.6%; *p* = 0.009). However, the distribution of AF subtypes did not differ significantly between the clusters. At baseline the proportion of NYHA functional class differed significantly between the two groups, with more patients in NYHA class III in Cluster 1 than in Cluster 2 (*p* = 0.031). Diabetes mellitus was also more common in Cluster 1 (47.0% vs. 23.3%; *p* = 0.004).

Patients in Cluster 1 had higher NT-proBNP concentrations than those in Cluster 2 (1431.03 ± 1295.64 vs. 383.34 ± 275.64 pg/mL; *p* < 0.001). In addition, Cluster 1 had a higher CHA_2_DS_2_VA score (5.12 ± 1.36 vs. 4.20 ± 1.52; *p* < 0.001). Overall, Cluster 1 was characterized by older age, a higher burden of atrial fibrillation, diabetes, and baseline clinical disease severity (Table 2).

### 3.5. External Functional and Patient-Reported Characteristics

Variables not included in cluster generation demonstrated clinically relevant differences between the two phenotypes. Cluster 1 showed consistently poorer functional capacity than Cluster 2. The six-minute walking distance was shorter in Cluster 1 (375.40 ± 109.72 vs. 430.57 ± 115.72 m; *p* = 0.004). Ventilatory efficiency was also impaired, with a higher minute ventilation/carbon dioxide production slope (VE/VCO_2_ slope) in Cluster 1 (39.43 ± 9.86 vs. 35.16 ± 7.86; *p* = 0.006). Peak oxygen uptake (VO_2_) was lower in Cluster 1 (1.11 ± 0.39 vs. 1.32 ± 0.43 L/min; *p* = 0.003) (Figure 5A). The corresponding standardized effect sizes were moderate (g = −0.48 for six-minute walking distance, 0.48 for VE/VCO_2_ slope, and −0.51 for peak VO_2_).

Quality-of-life categories differed significantly between clusters. Good quality of life was reported by 30.8% of patients in Cluster 1 compared with 61.6% in Cluster 2, whereas moderate or poor quality of life was more frequent in Cluster 1 (*p* = 0.003) (Figure 5B). In ordinal logistic regression, Cluster 1 was associated with higher odds of belonging to a worse quality-of-life category (OR 2.95; 95% CI 1.48–5.89, *p* = 0.002) (Figure 6).

The total Minnesota Living with Heart Failure Questionnaire (MLHFQ) score was also higher in Cluster 1 (31.58 ± 17.70 vs. 22.03 ± 16.95; *p* = 0.003; g = 0.55). After Benjamini-Hochberg correction across the 21 individual MLHFQ items, six item-level differences remained statistically significant: need for daytime rest, difficulty with housework or gardening, social limitations, dyspnoea, fatigue, and heart-failure-related worry (all q ≤ 0.029; rank-biserial r = 0.27–0.34) (Table 3 and Supplementary Table S3).

### 3.6. Follow-Up Clinical Outcomes

During 12-month follow-up, at least one all-cause hospitalization occurred in 32.7% of patients in Cluster 1 compared with 15.0% in Cluster 2 (*p* = 0.047) (Figure 7A). Given the limited number of events and baseline differences between clusters, these analyses were considered exploratory and should not be interpreted as evidence of prognostic value. No statistically significant differences were observed for general practitioner visits (*p* = 0.874). Cardiologist visits were numerically more frequent in Cluster 1 (56.9% vs. 38.3%; *p* = 0.079), but the association did not reach statistical significance (OR 2.04; 95% CI 0.97–4.31; *p* = 0.061). Changes in NYHA class from baseline to follow-up did not differ significantly between clusters. Improvement was observed in 11.3% of Cluster 1 and 23.3% of Cluster 2, while worsening occurred in 15.1% and 11.7%, respectively (*p* = 0.241) (Figure 7B). In an ordinal logistic regression adjusted for baseline NYHA class, Cluster 1 showed numerically higher odds of worse NYHA class at follow-up, although this did not reach statistical significance (OR 2.01; 95% CI 0.89–4.52; *p* = 0.093) (Figure 6) (Table 4).

### 3.7. Atrial and Electrical Characteristics

#### 3.7.1. Structural and Hemodynamic Characteristics

As expected from the cluster-defining profile, LAVI and TR pressure gradient differed between the two groups. Because both variables contributed directly to cluster generation, these differences were considered defining characteristics of the phenotypes rather than independent evidence supporting the cluster solution. Among additional echocardiographic measures not included directly in the clustering algorithm, left atrial end-systolic volume was higher in Cluster 1 than in Cluster 2 (98.55 ± 43.22 vs. 73.63 ± 19.29 mL; *p* < 0.001). Systolic pulmonary artery pressure (sPAP) was likewise higher, although measurements were only available in a smaller subset of patients (Table 5).

#### 3.7.2. Functional Atrial Remodeling

Reduced four-chamber LA reservoir strain was a defining characteristic of Cluster 1 because LASr was included directly in cluster generation. Accordingly, its between-cluster difference is not interpreted as independent validation of the phenotype.

LA conduit strain and contraction strain were also reduced in Cluster 1, with four-chamber conduit strain of −9.13 ± 4.09% versus −11.40 ± 4.89% (*p* = 0.004) and contraction strain, measured only in SR during echocardiography because it is rhythm-dependent, of −5.58 ± 4.45% versus −10.12 ± 6.72% (*p* < 0.001). The derived LA stiffness index was also higher in Cluster 1 (1.65 ± 1.55 vs. 0.74 ± 0.49; *p* < 0.001). Because LA stiffness was calculated from mean E/e′ and the absolute value of LASr, both of which contributed to cluster generation, this difference was considered descriptive characterization of the phenotype rather than an independent validating feature. Patients in Cluster 1 also exhibited higher transmitral E-wave velocity. Mean E/e′ also differed between clusters, as expected because it was included as a cluster-defining variable. In contrast, neither peak LASr nor the absolute change in LASr differed significantly between groups (*p* = 0.102 and *p* = 0.951). These exercise-derived findings should be considered exploratory because of the high proportion of missing measurements (Table 5). Measurement availability decreased substantially at higher workloads in stress echo. For four-chamber LASr, measurements at 75 W were available in 13/66 patients in Cluster 1 and 22/87 patients in Cluster 2, and at 100 W in only 3/66 and 12/87 patients, respectively. Accordingly, workload-specific comparisons at these advanced exercise stages were considered exploratory (Supplementary Table S2). To assess the potential influence of rhythm during echocardiographic acquisition, a sensitivity analysis was performed in the 108 patients who were in sinus rhythm during resting echocardiography (Cluster 1, n = 38; Cluster 2, n = 70). Between-cluster differences in LASr, LAVI, LA stiffness, and LA contraction strain remained significant in the sinus-rhythm subgroup. The differences in peak VO_2_, VE/VCO_2_ slope, six-minute walking distance, and MLHFQ score were also preserved in this subgroup. Full results are provided in Supplementary Table S6.

#### 3.7.3. Electrical Atrial Remodeling

In contrast to the structural and functional variables used for cluster generation, the electrical parameters described below were not included in the clustering algorithm and therefore provide additional characterization of the phenotypes.

Electrical atrial characteristics also differed between clusters. The resting ECG rhythm distribution differed markedly, with SR present in 54.5% of Cluster 1 and 89.7% of Cluster 2, while AF was present in 34.8% and 8.0%, respectively (*p* < 0.001). Resting heart rate was higher in Cluster 1 (71.61 ± 15.95 vs. 64.87 ± 11.64 bpm; *p* = 0.005). Among P-wave parameters, P-wave vector in the precordial plane was significantly greater in Cluster 1 than in Cluster 2 (68.32 ± 38.20° vs. 48.84 ± 30.72°; *p* = 0.021) and P-wave amplitude in lead I was lower in Cluster 1 than in Cluster 2 (0.05 ± 0.04 vs. 0.08 ± 0.04; *p* = 0.007). Other P-wave characteristics, including maximum P-wave duration, P-wave dispersion, P-wave area in lead II, P-terminal force in V1, and the prevalence of interatrial block, did not differ significantly between clusters (Table 5).

## 4. Discussion

### 4.1. Main Findings

In this study, we applied an unsupervised consensus clustering approach to a well-characterized HFpEF cohort and identified a reproducible two-cluster solution. Our analysis resulted in four main observations. First, the clusters were defined by distinct multidimensional profiles across the prespecified input variables, including atrial structure and mechanics, haemodynamic surrogates, biomarkers, renal function, and metabolic characteristics. Because these measures contributed directly to cluster generation, their between-cluster separation represents phenotype definition rather than independent validation. Second, the clusters also differed in clinically relevant variables that were not included in the clustering algorithm. Cluster 1 showed lower peak VO_2_, poorer ventilatory efficiency, shorter six-minute walking distance, and worse health-related quality of life. Third, Cluster 1 showed a higher observed hospitalization burden. Because of the limited numbers of events, this finding should not be interpreted as evidence of independent prognostic value. Finally, sensitivity analyses supported the robustness of the principal cluster structure across alternative analytical conditions, including exclusion of individual clustering variables and complete-case analyses.

### 4.2. Multidimensional Atrial Remodeling Beyond Atrial Size Alone

There is increasing evidence that the LA plays a central role in the pathophysiology of HFpEF. Although left atrial enlargement is an established marker for chronically elevated left ventricular filling pressures and disease severity, our findings suggest that atrial dysfunction provides additional clinically relevant information beyond atrial size alone.

Patients in Cluster 1 had significantly larger left atrial volumes than those in Cluster 2. Both clusters showed left atrial enlargement. According to current echocardiographic reference ranges, the mean LAVI for Cluster 2 (38.8 mL/m^2^) corresponds to mild left atrial enlargement, whilst the mean LAVI for Cluster 1 (53.1 mL/m^2^) is in the range of severe enlargement. Despite this difference in severity, a significant overlap in LAVI values between the clusters was observed (Figure 4). Furthermore, excluding LAVI from the clustering model resulted in only a slight reduction in cluster stability, with more than 90% of patients remaining in their original cluster classification. In contrast, functional measures of atrial remodeling, particularly left atrial reservoir strain and left atrial stiffness, were markedly impaired in the biomarker-high, atrial-dysfunction phenotype. Patients with the biomarker-high, atrial-dysfunction phenotype exhibited marked impairment of atrial function, which was associated with higher left ventricular filling pressures, elevated pulmonary pressures, and higher concentrations of natriuretic peptides and reduced renal function. These findings suggest that the identified phenotype is characterized not primarily by the size of the LA, but by advanced atrial remodeling, which combines structural changes with functional impairment and biochemical evidence of increased cardiac and organ burden.

The clinical relevance of the phenotype was more directly supported by associations with variables not used for cluster generation. Patients in Cluster 1 exhibited greater functional impairment in the 6 min walk test and exercise testing (lower peak VO_2_ and higher VE/VCO_2_ slope), and a more unfavorable pattern of healthcare utilization, with a higher observed all-cause hospitalization burden. In HFpEF, peak VO_2_ provides an objective measure of aerobic exercise capacity, whereas the VE/VCO_2_ slope reflects ventilatory efficiency and may provide complementary information on the mechanisms underlying exercise intolerance. These findings are consistent with previous evidence linking impaired left atrial function to reduced exercise capacity in heart failure [24,25]. Together, these associations support the clinical relevance of atrial dysfunction as a component of an HFpEF phenotype with a greater overall disease burden.

AF is common in HFpEF and is associated with increased morbidity and mortality. However, the relationship between AF and atrial remodeling is complex and bidirectional as structural remodeling predisposes to AF while AF itself accelerates functional deterioration of the atria [8,9,10,25,26]. In our cohort, AF was more common in the biomarker-high, atrial-dysfunction cluster. However, the differences between the clusters were not explained solely by rhythm status at the time of echocardiography, but extended to atrial mechanics, filling and pulmonary pressures, renal function, biomarkers, exercise capacity, and healthcare utilization.

Taken together, these observations support the concept that atrial myopathy and AF are related but not interchangeable entities and suggest that the identified phenotype captures features beyond rhythm status alone [27,28]. Whether advanced atrial remodeling is simply a marker of disease burden or actively contributes to the progression of HFpEF cannot be determined from this observational study.

### 4.3. Comparison with Previous Studies

The present findings are consistent with the growing recognition that HFpEF is not a single disease entity but rather a heterogeneous syndrome including multiple biological and clinical phenotypes. The seminal phenomapping study by Shah et al. demonstrated for the first time that unsupervised machine learning techniques can identify reproducible HFpEF phenogroups with distinct clinical characteristics, cardiac structure, biomarker profiles, exercise capacity, and prognosis, thereby establishing the concept of phenotype-based risk stratification in HFpEF [26]. Subsequent studies confirmed these observations across different HFpEF cohorts and highlighted the potential of phenomapping to improve biological understanding and future precision medicine approaches [29,30]. However, most previous clustering studies primarily incorporated conventional clinical characteristics and standard echocardiographic variables, while atrial disease was generally represented by AF status or left atrial size rather than by a comprehensive assessment of atrial remodeling. Our study extends these observations by incorporating detailed measures of atrial structure and mechanics into a multidimensional phenotyping approach and identifying a distinct biomarker-high, atrial-dysfunction phenotype within this cohort.

Our findings closely align with the concept of disproportionate left atrial myopathy described by Patel et al. in the PROMIS-HFpEF study [31]. In that study, patients with disproportionate atrial myopathy exhibited impaired left atrial reservoir function despite relatively preserved left ventricular mechanics and were characterized by higher pulmonary artery pressures, increased pulmonary vascular resistance, reduced stroke volume reserve, and a distinct proteomic profile that was not fully explained by AF [31]. The atrial, haemodynamic, biomarker, and renal characteristics primarily describe the defining profile of the cluster because several of these variables contributed directly to cluster generation. Importantly, the identified biomarker-high, atrial-dysfunction phenotype was also associated with clinically relevant measures that were not included in the clustering algorithm, including impaired exercise capacity and poorer quality of life. These clinical associations support the clinical relevance of the biomarker-high, atrial-dysfunction phenotype, while the present observational data do not establish that atrial dysfunction represents an independent pathophysiological process beyond ventricular dysfunction or AF.

Beyond HFpEF, the relevance of LA strain as a marker of atrial functional impairment has also been demonstrated in other cardiomyopathic conditions, including cardiac amyloidosis and hypertrophic cardiomyopathy, underscoring the complementary information provided by speckle-tracking-derived atrial deformation measures [32].

An important extension of previous work is the difference between atrial enlargement and atrial dysfunction. LAVI is well established as an integrated marker of chronically elevated left ventricular filling pressures and cumulative atrial remodeling [26,31]. In our cohort, the biomarker-high, atrial-dysfunction phenotype was characterized by severe left atrial enlargement, whereas patients in Cluster 2 exhibited only mild enlargement according to current echocardiographic reference values. Nevertheless, substantial overlap of individual LAVI values was observed, and exclusion of LAVI from the clustering algorithm resulted in only a modest reduction in cluster stability. Functional atrial measures were prominent components of the identified phenotype. However, because LASr contributed directly to cluster generation and LA stiffness is derived in part from cluster-defining variables, these between-cluster differences should not be interpreted as independent evidence of superiority over structural measures. Rather, the LAVI-exclusion sensitivity analysis suggests that atrial size alone did not account for the observed cluster structure.

The growing importance of atrial function in HFpEF has also been emphasized in recent mechanistic studies demonstrating that impaired left atrial reservoir strain is associated with elevated exercise filling pressures, reduced cardiac reserve, impaired peak oxygen uptake, and an increased risk of heart failure hospitalization and mortality [27,31]. Our findings are highly consistent with these observations. Patients belonging to the biomarker-high, atrial-dysfunction phenotype exhibited significantly lower peak VO_2_, shorter six-minute walk distance, poorer quality of life, and greater healthcare utilization. Thus, our results extend previous literature by demonstrating that advanced atrial remodeling is reflected not only by adverse hemodynamic but also by a considerably higher clinical disease burden.

The relationship between AF and atrial remodeling remains complex and bidirectional. Previous studies have shown that atrial remodeling promotes the development and maintenance of AF, whereas AF itself accelerates structural and functional deterioration of the atrium [26,31]. In the PROMIS-HFpEF study, disproportionate atrial myopathy remained associated with adverse haemodynamic and a distinct biological profile even after accounting for AF, suggesting that atrial myopathy represents a disease entity beyond rhythm status [31]. Likewise, although AF was more prevalent in the biomarker-high, atrial-dysfunction phenotype in our cohort, the identified clusters differed simultaneously. These observations further support the concept that AF and atrial myopathy should not be considered interchangeable entities.

Finally, our findings may also have therapeutic implications. The EAST-AFNET 4 trial demonstrated that early rhythm-control therapy reduces cardiovascular outcomes in patients with recently diagnosed AF, including those with heart failure, the majority of whom had preserved left ventricular ejection fraction [33]. However, this strategy comprised both antiarrhythmic drug therapy and catheter ablation and therefore does not establish whether catheter ablation is equally effective across different HFpEF phenotypes. Moreover, a recent meta-analysis of randomized trials did not demonstrate a clear reduction in heart failure events following catheter ablation in patients with HFpEF, although the available evidence remains limited and heterogeneous [34]. Conversely, observational studies suggest that left atrial reservoir strain and left atrial stiffness are associated with atrial fibrosis, low-voltage substrate, and AF recurrence after catheter ablation, indicating that the extent of atrial remodeling may influence procedural success [35].

Taken together, these observations support the hypothesis that atrial remodeling may represent one of several factors associated with the response to rhythm-control strategies. However, the present study was not designed to evaluate treatment effects, and whether atrial phenotyping can identify patients who derive differential benefit from specific rhythm-control approaches requires prospective validation. Multidimensional HFpEF phenotyping may therefore provide a framework for investigating whether the degree of atrial remodeling is associated with differential responses to rhythm-control interventions, including catheter ablation.

### 4.4. Clinical Relevance and Future Directions

The present findings suggest that multidimensional phenotyping may improve characterization of disease burden in HFpEF. In particular, the association of cluster membership with exercise limitation and impaired quality of life indicates that the identified phenotypes capture clinically relevant differences beyond the variables used to generate the clusters.

Patients in Cluster 1 showed a greater impairment in quality of life and physical performance, as well as a higher burden in terms of healthcare utilization. Whether patients with this phenotype benefit from more intensive outpatient follow-up or phenotype-guided management requires prospective evaluation.

Furthermore, our findings could inform the design of future randomized trials. The neutral outcomes of previous HFpEF trials have been attributed in part to the biological heterogeneity of the syndrome and phenotype-based enrichment. It has been proposed that randomizing or stratifying patients according to data-driven phenogroups, rather than broad inclusion criteria alone, would increase statistical power and target therapies at the patients most likely to respond [36,37]. Whether the degree of atrial remodeling modifies the clinical benefit of interventions such as catheter ablation remains unknown. The biomarker-high, atrial-dysfunction phenotype identified here may provide a candidate for evaluation in future phenotype-enriched clinical trials.

Impaired left atrial reservoir strain provides incremental value for the prediction of new-onset AF in HFpEF beyond clinical risk factors and left atrial volume [38,39] and continuous rhythm monitoring reveals a substantial burden of previously undetected AF in HFpEF or mildly reduced ejection fraction [40]. Although untargeted screening with implantable loop recorders detected approximately three times more AF in high-risk individuals in the LOOP trial without significantly reducing stroke rates [41], the clinical value of targeted rhythm surveillance in patients with advanced atrial remodeling remains uncertain and requires prospective evaluation.

### 4.5. Limitations

Several limitations of the present study should be acknowledged. It was a single-center observational study with a moderate sample size, which may limit the generalizability of our findings. In addition, patients with an LVEF of 40–49% were eligible for inclusion, so the cohort also comprises patients classified as heart failure with reduced ejection fraction under current guideline definitions; this should be considered when comparing our findings with strictly defined HFpEF populations [14]. However, the principal cluster structure and functional characteristics were preserved in the sensitivity analysis restricted to patients with LVEF ≥ 50%.

Although the identified phenotype showed high reproducibility across multiple imputations and the principal cluster structure was preserved in sensitivity analyses excluding LAVI, NT-proBNP, complete-case analysis, and restriction to patients with LVEF ≥ 50%. External validation in independent HFpEF cohorts is nevertheless required before clinical implementation. Additionally, the number of clinical outcome events, particularly hospitalizations, was relatively small, which limited statistical power for outcome analyses.

Hospitalization outcomes during the 12-month follow-up were primarily obtained by telephone interview and were not independently verified. Therefore, we limited the outcome analysis to all-cause hospitalization.

The clustering approach is exploratory and depends on the selection of input variables, preprocessing strategy, and clustering algorithm. While the selected variables were clinically motivated and the identified phenotype remained robust across multiple sensitivity analyses, alternative methodological approaches may yield different phenotypic classifications.

Furthermore, differences in variables used for cluster generation cannot be regarded as independent validation of the resulting phenotypes. We therefore distinguished these cluster-defining characteristics from external clinical variables.

Missing values in the ten baseline variables used for clustering, including several resting echocardiographic measures, were handled using multiple imputation by chained equations under a conditional missing-at-random assumption. This approach allowed all patients to contribute to the clustering procedure and avoided restricting the primary analysis to complete cases, although its validity depends on the underlying imputation model and missingness assumption. Stress-echocardiographic variables were not included in the imputation model and were analyzed based on available observations. Measurement availability decreased substantially at higher exercise stages, particularly at 75 and 100 W, making these workload-specific comparisons susceptible to selection bias. Accordingly, stress-echocardiographic findings at advanced exercise stages should be regarded as exploratory and hypothesis-generating.

No formal inter- or intra-observer reproducibility analysis was performed for the echocardiographic strain measurements, which should be considered when interpreting measurement-dependent differences. Additionally, left atrial stiffness was derived from echocardiographic measurements rather than from an invasive hemodynamic assessment. While this non-invasive index has been validated and is widely used as a surrogate marker of atrial compliance, it remains an indirect estimate of atrial mechanical properties [19].

Finally, the observational design precludes causal inference and does not permit conclusions regarding the therapeutic implications of the identified phenotype. Moreover, follow-up was limited to 12 months. Longer-term, prospective studies that include adjudicated cardiovascular outcomes, treatment response data, and external validation cohorts are necessary to determine the prognostic and therapeutic relevance of the identified atrial phenotype.

Taken together, the present findings should be considered hypothesis-generating and confirmed in larger, prospective, multicenter studies.

## 5. Conclusions

Using consensus clustering, we identified two multidimensional phenotypes defined by differences in atrial structure and mechanics, hemodynamic burden, biomarkers, renal function, and metabolic characteristics. Because these variables contributed directly to cluster generation, their between-cluster differences represent phenotype-defining features rather than independent validation. Importantly, Cluster 1 was also associated with poorer exercise capacity, reduced six-minute walking distance, worse health-related quality of life, and a higher observed hospitalization burden. These clinical associations suggest that the identified phenotype captures differences in overall disease burden, although prognostic analyses remain exploratory. The findings are hypothesis-generating and require external validation in independent prospective cohorts.

## Figures and Tables

**Figure 1:**
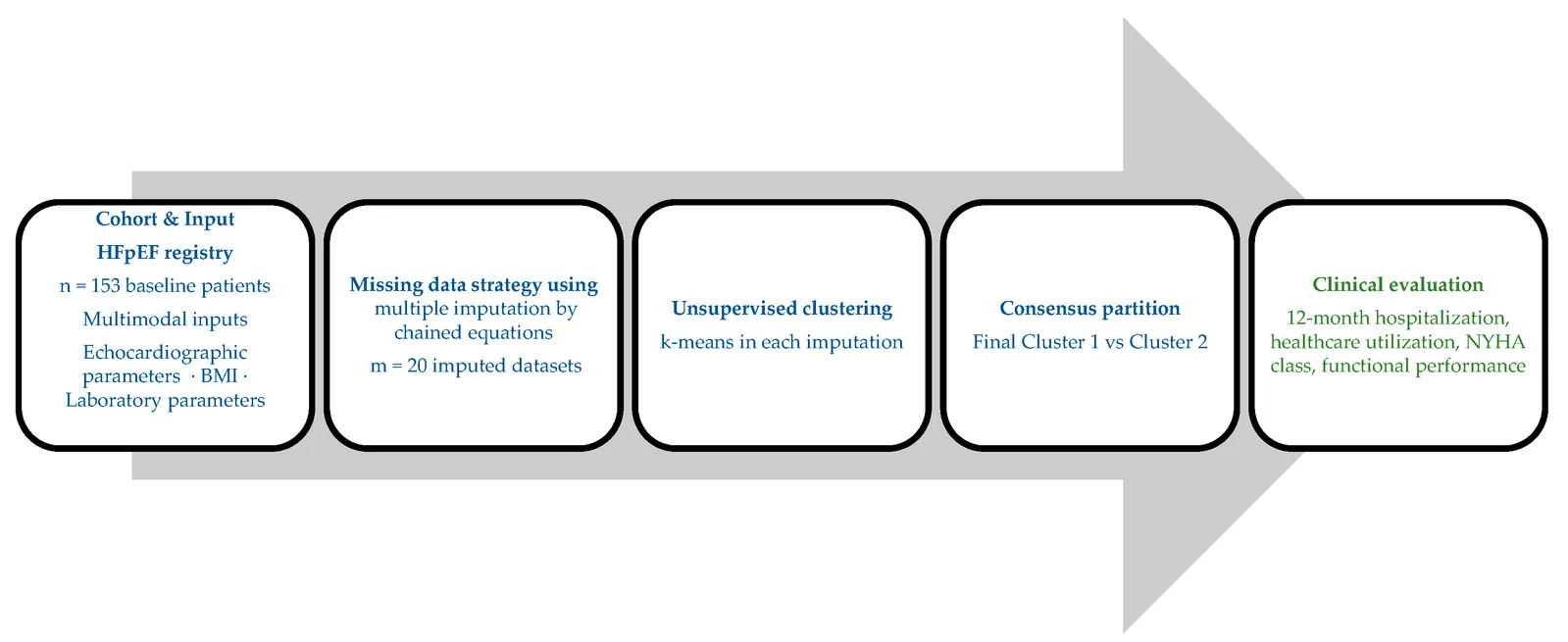
Analytical workflow of the clustering approach. Multimodal baseline data from 153 HFpEF registry patients were processed using multiple imputation by chained equations, followed by k-means clustering in each of 20 imputed datasets. Final cluster assignments were derived by consensus partitioning and evaluated with respect to 12-month hospitalization and healthcare utilization outcomes.

**Figure 2:**
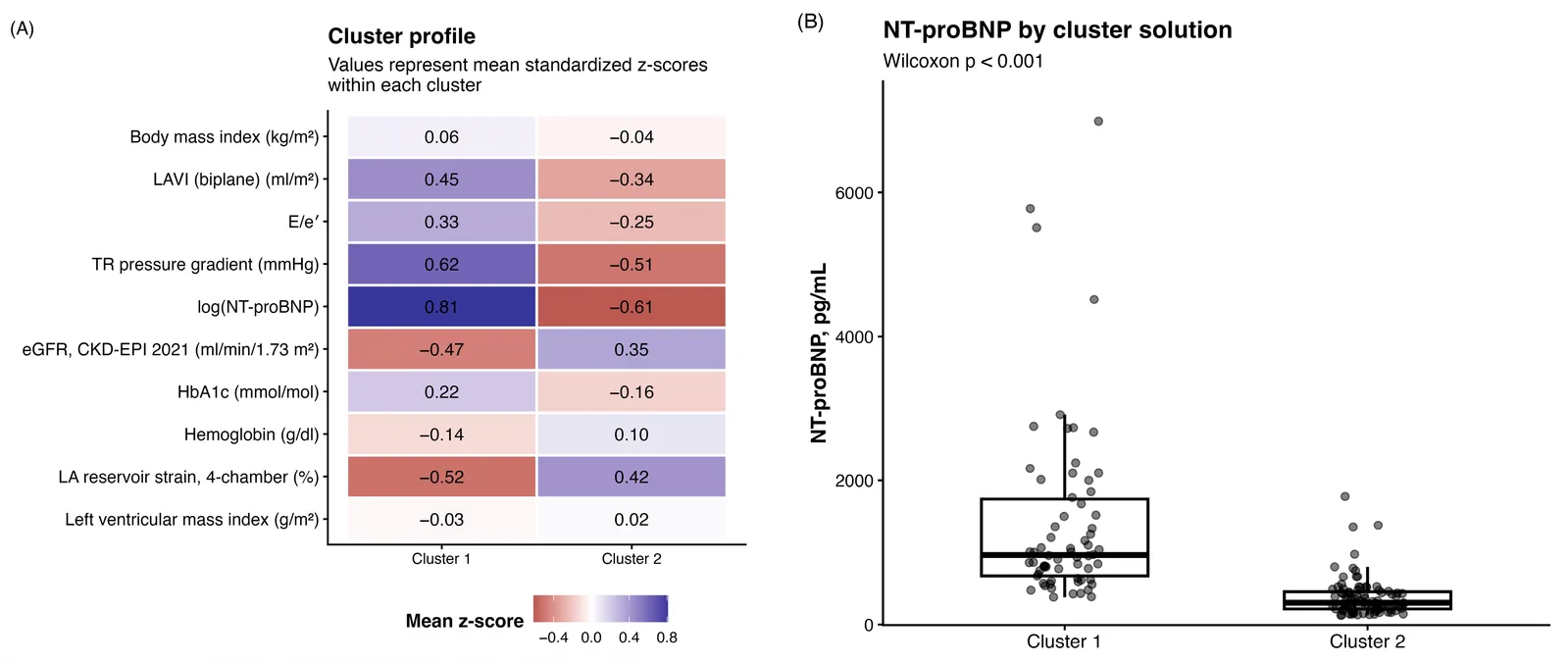
Standardized cluster profile of the Cluster solution and NT-proBNP according to cluster assignment. (**A**) Mean standardized (z-score) values for the ten variables used in the clustering procedure are presented for each consensus cluster. Positive z-scores represent values above the cohort mean and negative z-scores values below the cohort mean. (**B**) Boxplots show the median and interquartile range of NT-proBNP; individual patient values are displayed as points.

**Figure 3:**
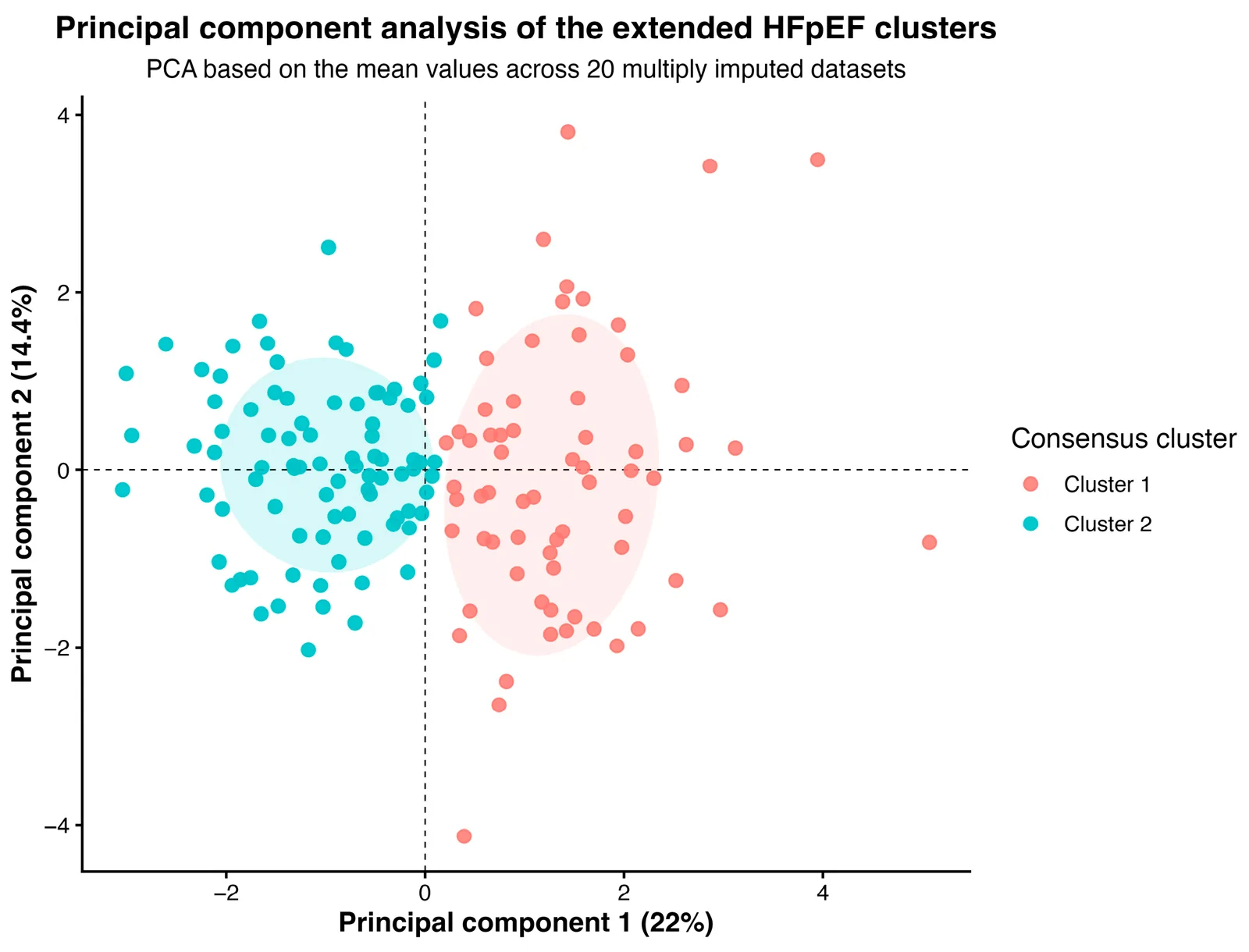
PCA of individuals by cluster assignment. Patients are projected onto the first two principal components and colored according to consensus cluster membership. Shaded ellipses represent the approximate distribution of each cluster. PCA was performed using patient-level mean values of the ten clustering variables across the 20 multiply imputed datasets after standardization. PCA was used for visualization only and did not contribute to cluster assignment.

**Figure 4:**
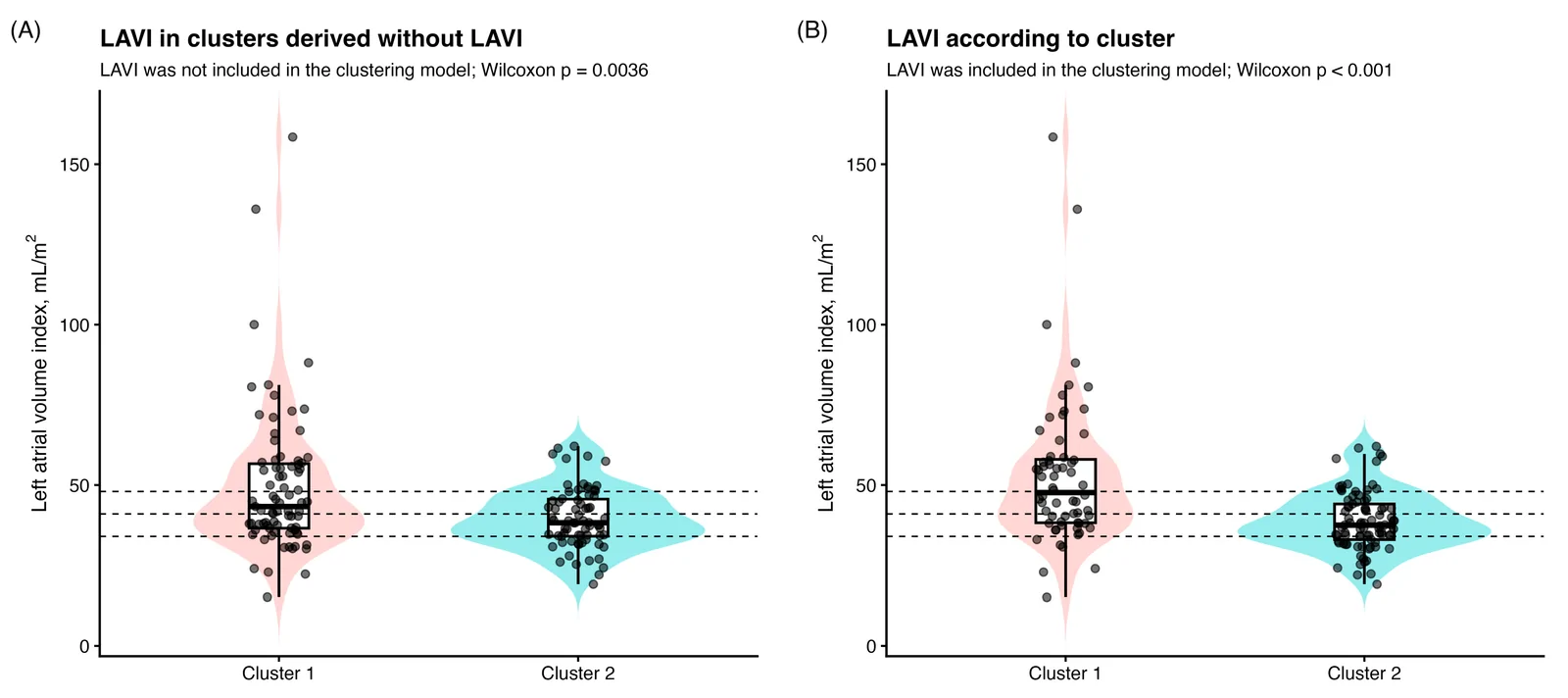
Left atrial volume index (LAVI) according to cluster assignment. (**A**) LAVI distribution in the sensitivity analysis excluding LAVI from the clustering model. (**B**) LAVI distribution in the clustering model; *p*-value is descriptive. Boxplots show the median and interquartile range, individual patient values are displayed as points. *p*-values are indicated in each panel.

**Figure 5:**
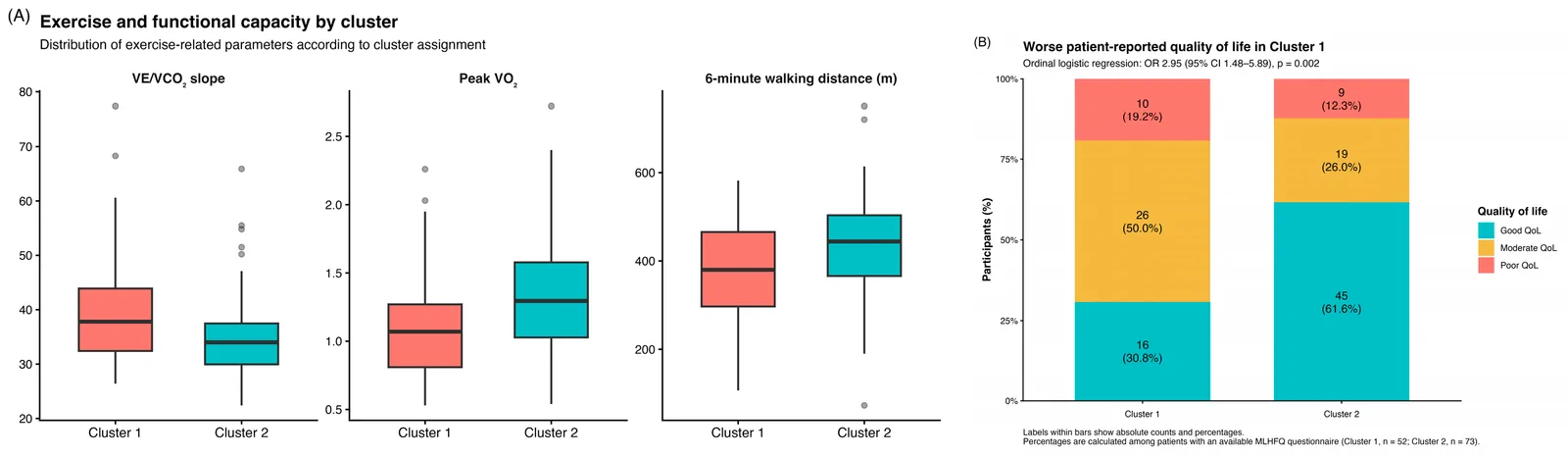
Quality of life and functional capacity according to consensus cluster membership. (**A**) VE/VCO_2_ slope, peak VO_2_, and six-minute walking distance according to cluster membership. Box plots display medians, interquartile ranges, and individual outlying observations. (**B**) Distribution of good, moderate, and poor quality-of-life categories according to clusters.

**Figure 6:**
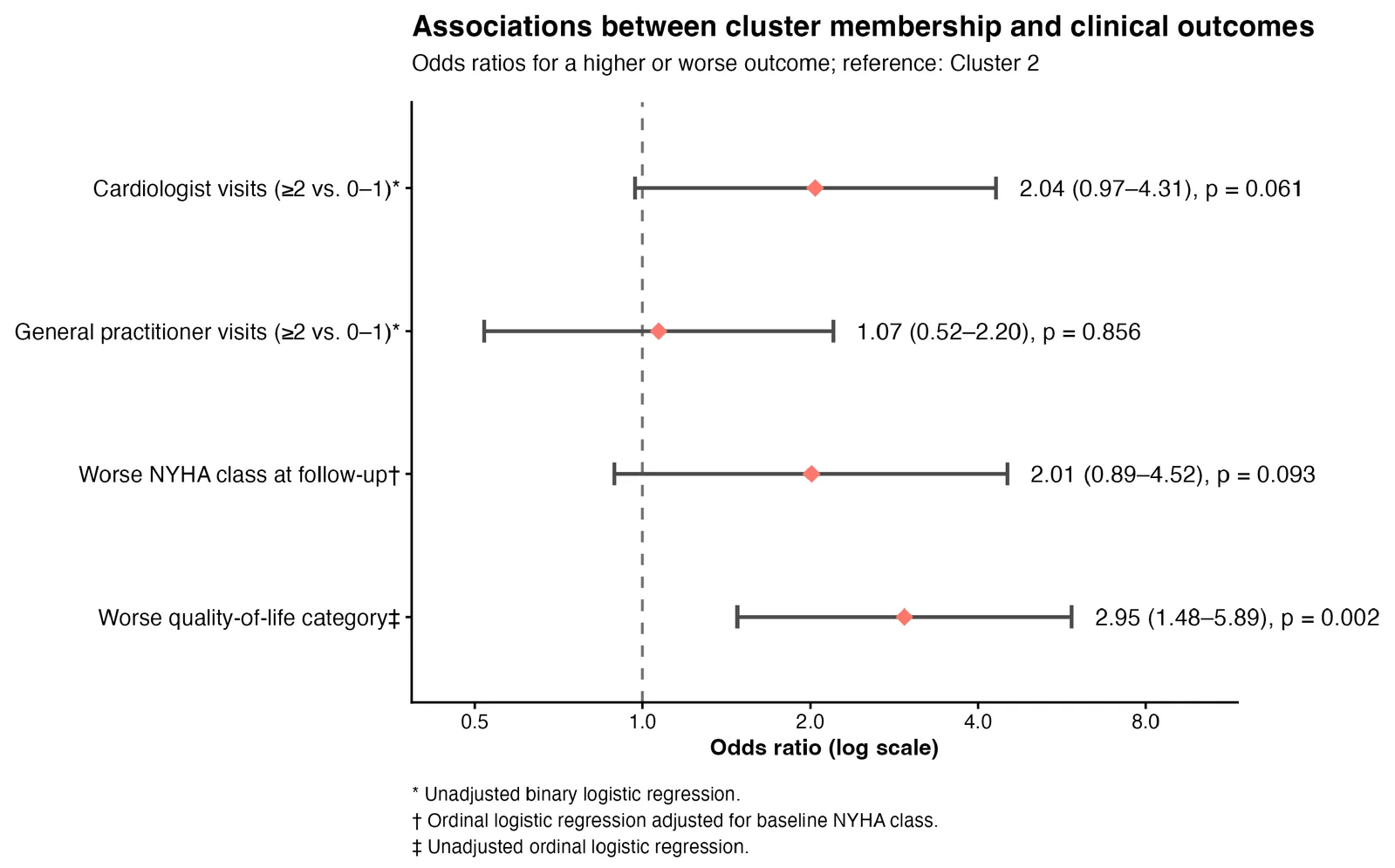
Association between cluster membership and clinical outcomes. Forest plot showing odds ratios (ORs) and 95% confidence intervals for the association between Cluster 1 (reference: Cluster 2) and categorized clinical outcomes. Odds ratios >1 indicate higher odds of a worse outcome category in Cluster 1. The dashed vertical line indicates no association (OR = 1). General practitioner and cardiologist visits were analyzed using unadjusted binary logistic regression. Quality-of-life categories were analyzed using unadjusted ordinal logistic regression, whereas follow-up NYHA class was analyzed using ordinal logistic regression adjusted for baseline NYHA class.

**Figure 7:**
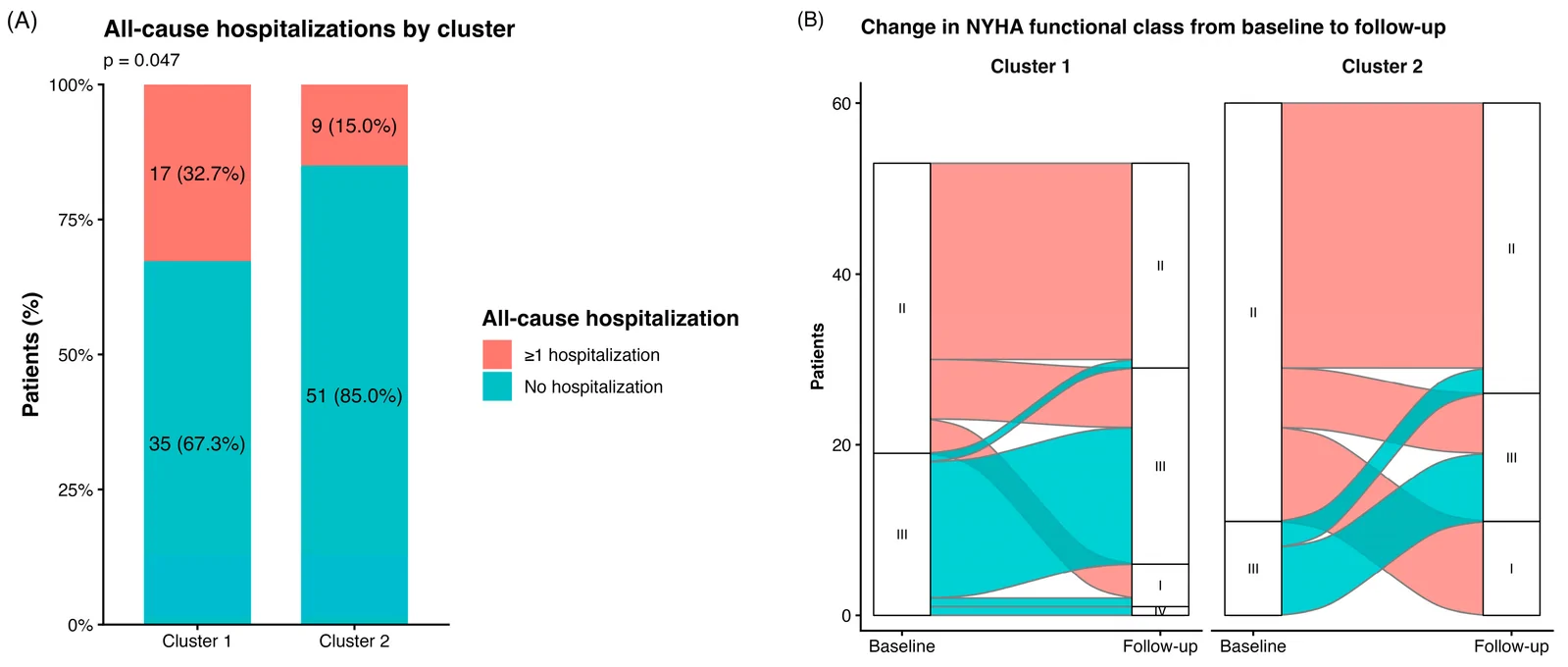
Changes in NYHA functional class and all-cause hospitalizations according to cluster assignment. (**A**) The panel shows the distribution of hospitalizations during follow-up, expressed as percentages within each cluster. (**B**) The panel shows individual transitions in NYHA functional class from baseline to follow-up for patients in Cluster 1 and Cluster 2. Flow widths correspond to the number of patients undergoing each transition; analyses were restricted to patients with available NYHA assessments at both time points.

**Table 1:** Distribution of cluster-defining variables according to consensus cluster membership. The values are presented as mean ± SD. Between-cluster comparisons were performed using Welch’s *t*-test. Descriptive values are based on the observed, non-imputed data.

Characteristics	Cluster 1 (N = 66)	Cluster 2 (N = 87)	*p*-Value
Body mass index (kg/m^2^)	28.74 ± 6.01	28.19 ± 4.65	0.542
LAVI (biplane) (mL/m^2^)	53.13 ± 23.55	38.82 ± 9.10	<0.001
E/e′	16.11 ± 8.40	12.38 ± 3.84	0.002
TR pressure gradient (mmHg)	30.65 ± 13.73	13.14 ± 12.19	<0.001
log (NT-proBNP)	7.01 ± 0.68	5.78 ± 0.56	<0.001
eGFR, CKD-EPI 2021 (mL/min/1.73 m^2^)	59.95 ± 17.86	75.27 ± 16.41	<0.001
HbA1c (mmol/mol)	42.80 ± 14.09	38.32 ± 9.40	0.028
Hemoglobin (g/dL)	13.11 ± 1.40	13.51 ± 1.90	0.130
LA reservoir strain, 4-chamber (%)	12.62 ± 6.27	20.66 ± 8.46	<0.001
Left ventricular mass index (g/m^2^)	109.10 ± 26.70	110.48 ± 29.50	0.769

**Table 2:** Baseline clinical Characteristics and Comorbidities. Baseline demographic, clinical and laboratory characteristics are shown for patients in Cluster 1 and Cluster 2, including missing data counts and *p*-values. Numeric variables are shown as mean ± SD. Categorical variables are shown as n (%). *p*-values are calculated using Welch *t*-test for numeric variables and chi-square or Fisher’s exact test for categorical variables. Effect size is calculated using Hedges’ g for continuous variables and Cramér’s V for categorical variables. Variables used for cluster generation are presented separately in Table 1 and are not repeated here.

Baseline Parameters	Cluster 1	Cluster 2	Missing C1	Missing C2	*p* Value	Effect Size
Sex (female)	33 (50.0%)	38 (43.7%)	0/66	0/87	0.540	0.06
Age (years)	75.98 ± 5.69	70.98 ± 8.23	0/66	0/87	<0.001	0.69
History of AF			0/66	0/87	0.009	0.22
No AF	18 (27.3%)	43 (49.4%)				
AF	48 (72.7%)	44 (50.6%)				
Type of AF			22/66	49/87	0.134	0.22
paroxysmal	16 (36.4%)	21 (55.3%)				
permanent	9 (20.5%)	3 (7.9%)				
persistent	19 (43.2%)	14 (36.8%)				
Diabetes mellitus	31 (47.0%)	20 (23.3%)	0/66	1/87	0.004	0.25
Arterial hypertension	62 (93.9%)	75 (87.2%)	0/66	1/87	0.269	0.11
Hyperlipidaemia	50 (76.9%)	52 (61.2%)	1/66	2/87	0.061	0.17
Coronary artery disease	36 (55.4%)	46 (54.1%)	1/66	2/87	1.000	0.01
Cerebrovascular disease	15 (22.7%)	15 (17.6%)	0/66	2/87	0.568	0.06
NYHA class at baseline			0/66	0/87	0.031	0.19
II	41 (62.1%)	69 (79.3%)				
III	25 (37.9%)	18 (20.7%)				
Pacemaker	13 (19.7%)	15 (17.2%)	0/66	0/87	0.859	0.03
Implantable cardioverter-defibrillator	5 (7.6%)	4 (4.6%)	0/66	0/87	0.501	0.06
NT-proBNP (pg/mL)	1431.03 ± 1295.64	383.34 ± 275.64	0/66	0/87	<0.001	1.19
Creatinine (mg/dL)	1.20 ± 0.42	0.98 ± 0.23	0/66	0/87	<0.001	0.68
CHA_2_DS_2_VA score	5.12 ± 1.36	4.20 ± 1.52	1/66	1/87	<0.001	0.63

**Table 3:** Functional capacity and health status stratified by cluster. Functional capacity, cardiopulmonary exercise testing, and quality of life are shown for patients in Cluster 1 and Cluster 2, including missing data counts and *p*-values. Numeric variables are shown as mean ± SD. Categorical variables are shown as n (%). *p*-values are calculated using Welch’s *t*-test for continuous variables and the chi-square test or Fisher’s exact test for categorical variables.

Functional Outcomes	Cluster 1	Cluster 2	Missing C1	Missing C2	*p* Value	Effect Size
6 min walking distance (m)	375.40 ± 109.72	430.57 ± 115.72	3/66	1/87	0.004	−0.48
VE/VCO_2_ slope	39.43 ± 9.86	35.16 ± 7.86	5/66	7/87	0.006	0.48
Peak VO_2_ (L/min)	1.11 ± 0.39	1.32 ± 0.43	5/66	7/87	0.003	−0.51
MLHFQ: Questionnaire score	31.58 ± 17.70	22.03 ± 16.95	14/66	14/87	0.003	0.55
MLHFQ Quality of Life			14/66	14/87	0.003	0.31
Good QoL (<24 Pts.)	16 (30.8%)	45 (61.6%)				
Moderate QoL (24–45 Pts.)	26 (50.0%)	19 (26.0%)				
Poor QoL (>45 Pts.)	10 (19.2%)	9 (12.3%)				

**Table 4:** Clinical outcomes at follow-up stratified by cluster. Clinical status, healthcare utilization, all-cause hospitalization measures, and follow-up NYHA class are shown for patients in Cluster 1 and Cluster 2, including missing data counts and *p*-values. Numeric variables are shown as mean ± SD. Categorical variables are shown as n (%). *p*-values are calculated using Welch’s *t*-test for continuous variables and the chi-square test or Fisher’s exact test for categorical variables. Effect sizes are reported as Hedges’ g for continuous variables and Cramér’s V for categorical variables.

Clinical Outcomes at Follow-Up	Cluster 1	Cluster 2	Missing C1	Missing C2	*p* Value	Effect Size
Status			12/66	27/87	0.272	0.16
Alive, telephone follow-up	48 (88.9%)	53 (88.3%)				
Alive, in-person follow-up	4 (7.4%)	7 (11.7%)				
deceased	2 (3.7%)	0 (0.0%)				
All-cause hospitalizations			14/66	27/87	0.047	0.21
≥1 Hospitalizations	17 (32.7%)	9 (15.0%)				
0 Hospitalizations	35 (67.3%)	51 (85.0%)				
General practitioner visits			16/66	28/87	0.874	0.04
≥2 Visits	43 (86.0%)	49 (83.1%)				
0–1 Visits	7 (14.0%)	10 (16.9%)				
Cardiologist visits			15/66	27/87	0.079	0.19
≥2 Visits	29 (56.9%)	23 (38.3%)				
0–1 Visits	22 (43.1%)	37 (61.7%)				
NYHA class at follow-up			13/66	27/87	0.079	0.24
I	5 (9.4%)	11 (18.3%)				
II	24 (45.3%)	34 (56.7%)				
III	23 (43.4%)	15 (25.0%)				
IV	1 (1.9%)	0 (0.0%)				
Changes in NYHA class			13/66	27/87	0.241	0.16
Improved	6 (11.3%)	14 (23.3%)				
Unchanged	39 (73.6%)	39 (65.0%)				
Worsened	8 (15.1%)	7 (11.7%)				
General practitioner visits (n)	6.05 ± 5.88	4.94 ± 4.45	15/66	28/87	0.274	0.21
Cardiologist visits (n)	1.85 ± 1.85	1.52 ± 2.28	15/66	27/87	0.404	0.16
All-cause hospitalizations (n)	0.48 ± 0.80	0.28 ± 0.92	14/66	27/87	0.229	0.23
Total days hospitalized (d)	3.92 ± 5.90	2.14 ± 4.63	16/66	28/87	0.086	0.34

**Table 5:** Atrial remodeling parameters stratified by cluster. Structural, functional, and electrical atrial remodeling parameters are shown for patients in Cluster 1 and Cluster 2, including missing data counts and *p*-values. LA contraction strain was analyzed only in patients in sinus rhythm during echocardiographic acquisition; measurements were available in 37/38 patients in Cluster 1 and 67/70 patients in Cluster 2. Numeric variables are shown as mean ± SD. Categorical variables are shown as n (%). *p*-values are calculated using Welch’s *t*-test for continuous variables and the chi-square test or Fisher’s exact test for categorical variables. Effect sizes are reported as Hedges’ g for continuous variables and Cramér’s V for categorical variables.

Atrial Parameters	Cluster 1 n = 66	Cluster 2 n = 87	Missing		*p* Value	Effect Size
			C1	C2		
Structural remodeling						
LA volume index, biplane (LAVI) (mL/m^2^)	53.13 ± 23.55	38.82 ± 9.10	2/66	2/87	<0.001	0.84
LA end-systolic volume (LAVES) (mL)	98.55 ± 43.22	73.63 ± 19.29	10/66	13/87	<0.001	0.78
Baseline TR Vmax (m/s)	2.65 ± 0.80	1.38 ± 1.18	8/66	16/87	<0.001	1.23
TR pressure gradient (mmHg)	30.65 ± 13.73	13.14 ± 12.19	8/66	16/87	<0.001	1.35
sPAP (mmHg)	41.13 ± 16.07	28.75 ± 6.91	27/66	71/87	<0.001	0.87
Functional remodeling						
LA reservoir strain, 4-chamber (%)	12.62 ± 6.27	20.66 ± 8.46	6/66	13/87	<0.001	−1.06
LA conduit strain, 4-chamber (%)	−9.13 ± 4.09	−11.40 ± 4.89	6/66	13/87	0.004	0.50
LA contraction strain, 4-chamber only in SR-Echo (%)	−5.58 ± 4.45	−10.12 ± 6.72	1/38	3/70	<0.001	0.75
LA stiffness index at baseline resting echo	1.65 ± 1.55	0.74 ± 0.49	9/66	17/87	<0.001	0.81
E/e′	16.11 ± 8.40	12.38 ± 3.84	3/66	4/87	0.002	0.60
Peak LA reservoir strain, 4-chamber (%)	19.29 ± 10.37	23.92 ± 11.40	42/66	47/87	0.102	−0.41
Absolute change in LA reservoir strain, 4-chamber (%)	4.64 ± 9.80	4.80 ± 10.61	42/66	48/87	0.951	−0.02
Electrical remodeling						
Heart rate on ECG (bpm)	71.61 ± 15.95	64.87 ± 11.64	0/66	0/87	0.005	0.49
ECG rhythm			0/66	0/87	<0.001	0.40
Atrial Tachycardia	2 (3.0%)	0 (0.0%)				
Sinus Rhythm	36 (54.5%)	78 (89.7%)				
Atrial flutter	5 (7.6%)	2 (2.3%)				
Atrial fibrillation	23 (34.8%)	7 (8.0%)				
Maximum P-wave duration (ms)	128.62 ± 23.26	122.54 ± 23.04	37/66	16/87	0.239	0.26
P-wave duration > 120 ms	23 (79.3%)	46 (64.8%)	37/66	16/87	0.235	0.14
P-wave dispersion (ms)	31.03 ± 21.93	33.46 ± 20.53	37/66	16/87	0.611	−0.12
Pathological P-wave dispersion	13 (44.8%)	32 (45.1%)	37/66	16/87	1.000	0.00
P-wave vector in the precordial plane (°)	68.32 ± 38.20	48.84 ± 30.72	38/66	17/87	0.021	0.59
Pathological P-wave vector	9 (32.1%)	14 (20.3%)	38/66	18/87	0.327	0.13
P-wave amplitude in lead I (mV)	0.05 ± 0.04	0.08 ± 0.04	37/66	16/87	0.007	−0.57
Pathological P-wave amplitude in lead I	29 (100.0%)	65 (91.5%)	37/66	16/87	0.177	0.16
P-wave area in lead II (mV*ms)	5.86 ± 2.53	6.23 ± 3.28	38/66	16/87	0.553	−0.12
Pathological P-wave area in lead II	22 (78.6%)	54 (76.1%)	38/66	16/87	0.998	0.03
P-terminal force in V1 (mV*ms)	2.61 ± 2.56	2.62 ± 2.30	37/66	16/87	0.977	−0.01
Pathological P-terminal force in V1	9 (31.0%)	20 (28.2%)	37/66	16/87	0.965	0.03
Biphasic P-wave in leads II, III and aVF	3 (10.3%)	6 (8.5%)	37/66	16/87	0.716	0.03
Interatrial block	3 (10.3%)	5 (7.2%)	37/66	18/87	0.691	0.05

## Data Availability

The data that support the findings of this study are available from the corresponding author upon reasonable request. The data are not publicly available due to privacy and ethical restrictions.

## Supplementary Materials

The following supporting information can be downloaded at: https://www.mdpi.com/article/10.3390/jcm15197538/s1, Supplementary Figure S1. Consensus clustering evaluation for the two-cluster solution. Supplementary Table S1: Resting echocardiographic characteristics stratified by cluster. Supplementary Table S2: Stress echocardiographic characteristics stratified by cluster. Supplementary Table S3: Minnesota Living with Heart Failure Questionnaire (MLHFQ) item scores stratified by cluster. Table S4: Missingness of the ten variables used for consensus clustering. Supplementary Table S5: Complete-case sensitivity analysis of the clustering solution. Supplementary Table S6: Sinus-rhythm sensitivity analysis. Supplementary Table S7: LVEF ≥ 50% sensitivity.

## Abbreviations

The following abbreviations are used in this manuscript:

AF	Atrial fibrillation
ARI	Adjusted Rand index
BMI	Body mass index
BP	Blood pressure
CHA_2_DS_2_-VA	Congestive heart failure, hypertension, age ≥ 75 years (2 points), diabetes mellitus, stroke/transient ischaemic attack/systemic embolism (2 points), vascular disease, and age 65–74 years
CI	Confidence interval
CKD-EPI 2021	Chronic Kidney Disease Epidemiology Collaboration 2021 equation
CPET	Cardiopulmonary exercise testing
E/e′	Ratio of early transmitral flow velocity to early diastolic mitral annular velocity
ECG	Electrocardiogram
eGFR	Estimated glomerular filtration rate
HbA1c	Glycated hemoglobin
HFpEF	Heart failure with preserved ejection fraction
IQR	Interquartile range
IVSED	Interventricular septal end-diastolic thickness
LA	Left atrium/left atrial
LAEF	Left atrial ejection fraction
LASr	Left atrial reservoir strain
LAVES	Left atrial end-systolic volume
LAVI	Left atrial volume index
LV	Left ventricular
LVEDD	Left ventricular end-diastolic diameter
LVEF	Left ventricular ejection fraction
LVMI	Left ventricular mass index
MLHFQ	Minnesota Living with Heart Failure Questionnaire
NSTEMI	Non-ST-elevation myocardial infarction
NT-proBNP	N-terminal pro-B-type natriuretic peptide
NYHA	New York Heart Association
OR	Odds ratio
PAC	Proportion of ambiguous clustering
PCA	Principal component analysis
PTFV1	P-terminal force in lead V1
PWED	Posterior wall end-diastolic thickness
QoL	Quality of life
QRS	QRS complex
QT	QT interval
RA	Right atrial
SD	Standard deviation
sPAP	Systolic pulmonary artery pressure
SR	Sinus rhythm
STEMI	ST-elevation myocardial infarction
SV	Supraventricular
TR	Tricuspid regurgitation
TR Vmax	Maximum tricuspid regurgitation velocity
VE/VCO_2_ slope	Minute ventilation/carbon dioxide production slope
Peak VO_2_	Peak oxygen uptake

## References

[1] Pfeffer M.A., Shah A.M., Borlaug B.A. (2019). Heart Failure with Preserved Ejection Fraction in Perspective. Circ. Res..

[2] Dunlay S.M., Roger V.L., Redfield M.M. (2017). Epidemiology of heart failure with preserved ejection fraction. Nat. Rev. Cardiol..

[3] Anker S.D., Usman M.S., Anker M.S., Butler J., Böhm M., Abraham W.T., Adamo M., Chopra V.K., Cicoira M., Cosentino F. (2023). Patient Phenotype Profiling in Heart Failure with Preserved Ejection Fraction to Guide Therapeutic Decision Making. A Scientific Statement of the Heart Failure Association, the European Heart Rhythm Association of the European Society of Cardiology, and the European Society of Hypertension. Eur. J. Heart Fail..

[4] Pecchia B., Samuel R., Shah V., Newman E., Gibson G.T. (2025). Mechanisms of exercise intolerance in heart failure with preserved ejection fraction (HFpEF). Heart Fail. Rev..

[5] Gan G.C.H., Ferkh A., Boyd A., Thomas L. (2018). Left atrial function: Evaluation by strain analysis. Cardiovasc. Diagn. Ther..

[6] Kupczyńska K., Mandoli G., Cameli M., Kasprzak J. (2021). Left atrial strain—A current clinical perspective. Pol. Heart J. (Kardiol. Pol.).

[7] Haykal R., Kassar A., Chamoun N., Akoum N. (2025). The left atrium in heart failure with preserved ejection fraction: What we know and what we do not know. Heart Rhythm O2.

[8] Fauchier L., Bisson A., Bodin A. (2023). Heart failure with preserved ejection fraction and atrial fibrillation: Recent advances and open questions. BMC Med..

[9] Kotecha D., Lam C.S., Van Veldhuisen D.J., Van Gelder I.C., Voors A.A., Rienstra M. (2016). Heart failure with preserved ejection fraction and atrial fibrillation: Vicious twins. J. Am. Coll. Cardiol..

[10] Ariyaratnam J.P., Elliott A.D., Mishima R.S., Gallagher C., Lau D.H., Sanders P. (2021). Heart failure with preserved ejection fraction: An alternative paradigm to explain the clinical implications of atrial fibrillation. Heart Rhythm O2.

[11] McDonagh T.A., Metra M., Adamo M., Gardner R.S., Baumbach A., Böhm M., Burri H., Butler J., Čelutkienė J., Chioncel O. (2021). 2021 ESC Guidelines for the diagnosis and treatment of acute and chronic heart failure. Eur. Heart J..

[12] Sanchis L., Gabrielli L., Andrea R., Falces C., Duchateau N., Perez-Villa F., Bijnens B., Sitges M. (2015). Left atrial dysfunction relates to symptom onset in patients with heart failure and preserved left ventricular ejection fraction. Eur. Heart J.-Cardiovasc. Imaging.

[13] Inoue K., Obokata M. (2025). Clinical utility of the left atrial strain analysis. J. Echocardiogr..

[14] Køber L., Adamo M., Ruwald A.-C., Tomasoni D., Anderson L.J., Andersson C., Brugts J.J., Chioncel O., Donal E., Ferdinandy P. (2026). 2026 ESC Guidelines for the management of heart failure: Developed by the task force for the management of heart failure of the European Society of Cardiology (ESC) with the special contribution of the Heart Failure Association (HFA) of the ESC Endorsed by the European Association for Cardio-Thoracic Surgery (EACTS). Eur. Heart J..

[15] Behlouli H., Feldman D.E., Ducharme A., Frenette M., Giannetti N., Grondin F., Michel C., Sheppard R., Pilote L. (2009). Identifying relative cut-off scores with neural networks for interpretation of the Minnesota Living with Heart Failure questionnaire. 2009 Annual International Conference of the IEEE Engineering in Medicine and Biology Society.

[16] Chen L.Y., Ribeiro A.L.P., Platonov P.G., Cygankiewicz I., Soliman E.Z., Gorenek B., Ikeda T., Vassilikos V.P., Steinberg J.S., Varma N. (2022). P Wave Parameters and Indices: A Critical Appraisal of Clinical Utility, Challenges, and Future Research—A Consensus Document Endorsed by the International Society of Electrocardiology and the International Society for Holter and Noninvasive Electrocardiology. Circ. Arrhythmia Electrophysiol..

[17] Mitchell C., Rahko P.S., Blauwet L.A., Canaday B., Finstuen J.A., Foster M.C., Horton K., Ogunyankin K.O., Palma R.A., Velazquez E.J. (2019). Guidelines for Performing a Comprehensive Transthoracic Echocardiographic Examination in Adults: Recommendations from the American Society of Echocardiography. J. Am. Soc. Echocardiogr..

[18] Javadi N., Bismee N.N., Abbas M.T., Scalia I.G., Pereyra M., Baba Ali N., Attaripour Esfahani S., Awad K., Farina J.M., Ayoub C. (2024). Left Atrial Strain: State of the Art and Clinical Implications. J. Pers. Med..

[19] Camilli M., Amore L., Ballacci F., Iannaccone G., Del Buono M.G., Giordano F., Graziani F., Sanna T., Pedicino D., Burzotta F. (2025). Echocardiographic left atrial stiffness index predicts high left ventricular filling pressures in patients with acute heart failure: An observational study. Clin. Res. Cardiol..

[20] Audigier V. clusterMI: Cluster Analysis with Missing Values by Multiple Imputation. https://cran.r-project.org/web/packages/clusterMI/vignettes/clusterMI.pdf.

[21] Resche-Rigon M., White I.R. (2018). Multiple imputation by chained equations for systematically and sporadically missing multilevel data. Stat. Methods Med. Res..

[22] Audigier V., Niang N. (2023). Clustering with missing data: Which equivalent for Rubin’s rules?. Adv. Data Anal. Classif..

[23] Harada D., Asanoi H., Noto T., Takagawa J. (2020). Different Pathophysiology and Outcomes of Heart Failure With Preserved Ejection Fraction Stratified by K-Means Clustering. Front. Cardiovasc. Med..

[24] Di Spigno F., Dall’Ospedale V., Gerra L., Breviario F., Tedeschi A., Trimarchi G., Aschieri D. (2025). Cardiopulmonary Exercise Testing and HFpEF: Diagnostic and Therapeutic Perspectives. Healthcare.

[25] Maffeis C., Rossi A., Cannata L., Zocco C., Belyavskiy E., Radhakrishnan A.K., Feuerstein A., Morris D.A., Pieske-Kraigher E., Pieske B. (2022). Left atrial strain predicts exercise capacity in heart failure independently of left ventricular ejection fraction. ESC Heart Fail..

[26] Shah S.J., Katz D.H., Selvaraj S., Burke M.A., Yancy C.W., Gheorghiade M., Bonow R.O., Huang C.-C., Deo R.C. (2015). Phenomapping for Novel Classification of Heart Failure With Preserved Ejection Fraction. Circulation.

[27] Reddy Y.N., Obokata M., Verbrugge F.H., Lin G., Borlaug B.A. (2020). Atrial dysfunction in patients with heart failure with preserved ejection fraction and atrial fibrillation. J. Am. Coll. Cardiol..

[28] Weerts J., Barandiarán Aizpurua A., Henkens M.T., Lyon A., van Mourik M.J., van Gemert M.R., Raafs A., Sanders-van Wijk S., Bayés-Genís A., Heymans S.R. (2022). The prognostic impact of mechanical atrial dysfunction and atrial fibrillation in heart failure with preserved ejection fraction. Eur. Heart J.-Cardiovasc. Imaging.

[29] Segar M.W., Patel K.V., Ayers C., Basit M., Tang W.H.W., Willett D., Berry J., Grodin J.L., Pandey A. (2020). Phenomapping of patients with heart failure with preserved ejection fraction using machine learning-based unsupervised cluster analysis. Eur. J. Heart Fail..

[30] Hedman Å.K., Hage C., Sharma A., Brosnan M.J., Buckbinder L., Gan L.M., Shah S.J., Linde C.M., Donal E., Daubert J.C. (2020). Identification of novel pheno-groups in heart failure with preserved ejection fraction using machine learning. Heart.

[31] Patel R.B., Lam C.S.P., Svedlund S., Saraste A., Hage C., Tan R.S., Beussink-Nelson L., Tromp J., Sanchez C., Njoroge J. (2021). Disproportionate left atrial myopathy in heart failure with preserved ejection fraction among participants of the PROMIS-HFpEF study. Sci. Rep..

[32] Monte I.P., Faro D.C., Trimarchi G., de Gaetano F., Campisi M., Losi V., Teresi L., Di Bella G., Tamburino C., de Gregorio C. (2023). Left Atrial Strain Imaging by Speckle Tracking Echocardiography: The Supportive Diagnostic Value in Cardiac Amyloidosis and Hypertrophic Cardiomyopathy. J. Cardiovasc. Dev. Dis..

[33] Kirchhof P., Camm A.J., Goette A., Brandes A., Eckardt L., Elvan A., Fetsch T., Gelder I.C.V., Haase D., Haegeli L.M. (2020). Early Rhythm-Control Therapy in Patients with Atrial Fibrillation. N. Engl. J. Med..

[34] Oraii A., McIntyre W.F., Parkash R., Kowalik K., Razeghi G., Benz A.P., Belley-Côté E.P., Conen D., Connolly S.J., Tang A.S.L. (2024). Atrial Fibrillation Ablation in Heart Failure with Reduced vs Preserved Ejection Fraction: A Systematic Review and Meta-Analysis. JAMA Cardiol..

[35] Marchandise S., Garnir Q., Scavée C., Varnavas V., le Polain de Waroux J.B., Wauters A., Beauloye C., Roelants V., Gerber B.L. (2022). Prediction of Left Atrial Fibrosis and Success of Catheter Ablation by Speckle Tracking Echocardiography in Patients Imaged in Persistent Atrial Fibrillation. Front. Cardiovasc. Med..

[36] Shah S.J. (2017). Innovative Clinical Trial Designs for Precision Medicine in Heart Failure with Preserved Ejection Fraction. J. Cardiovasc. Transl. Res..

[37] Oikonomou E.K., Thangaraj P.M., Bhatt D.L., Ross J.S., Young L.H., Krumholz H.M., Suchard M.A., Khera R. (2023). An explainable machine learning-based phenomapping strategy for adaptive predictive enrichment in randomized clinical trials. npj Digit. Med..

[38] Serenelli M., Cantone A., Dal Passo B., Di Ienno L., Fiorio A., Pavasini R., Passarini G., Bertini M., Campo G. (2023). Atrial Longitudinal Strain Predicts New-Onset Atrial Fibrillation: A Systematic Review and Meta-Analysis. JACC Cardiovasc. Imaging.

[39] Jasic-Szpak E., Marwick T.H., Donal E., Przewlocka-Kosmala M., Huynh Q., Gozdzik A., Woznicka A.K., Jankowska E.A., Ponikowski P., Kosmala W. (2021). Prediction of AF in Heart Failure with Preserved Ejection Fraction: Incremental Value of Left Atrial Strain. JACC Cardiovasc. Imaging.

[40] Gorter T.M., van Veldhuisen D.J., Mulder B.A., Artola Arita V.A., van Empel V.P.M., Manintveld O.C., Tieleman R.G., Maass A.H., Vernooy K., van Gelder I.C. (2023). Prevalence and Incidence of Atrial Fibrillation in Heart Failure with Mildly Reduced or Preserved Ejection Fraction: (Additive) Value of Implantable Loop Recorders. J. Clin. Med..

[41] Narducci M.L., Volpe M. (2022). Implantable recorders and systematic atrial fibrillation detection do not outperform standard of care in stroke prevention in the LOOP study. Eur. Heart J..

