# Supplementary material for: Cluster-Based Phenotyping in an HFpEF Population Identifies a Biomarker-High, Atrial-Dysfunction Phenotype with Impaired Quality of Life, Reduced Exercise Capacity and Higher Healthcare Utilization
Source: J Clin Med. 2026 Sep 28;15(19):7538. doi: 10.3390/jcm15197538 (PMC13666117; doi:10.3390/jcm15197538)
Supplement: Supplementary file 1 [file jcm-15-07538-s001.zip › jcm-4571877-supplementary.pdf]

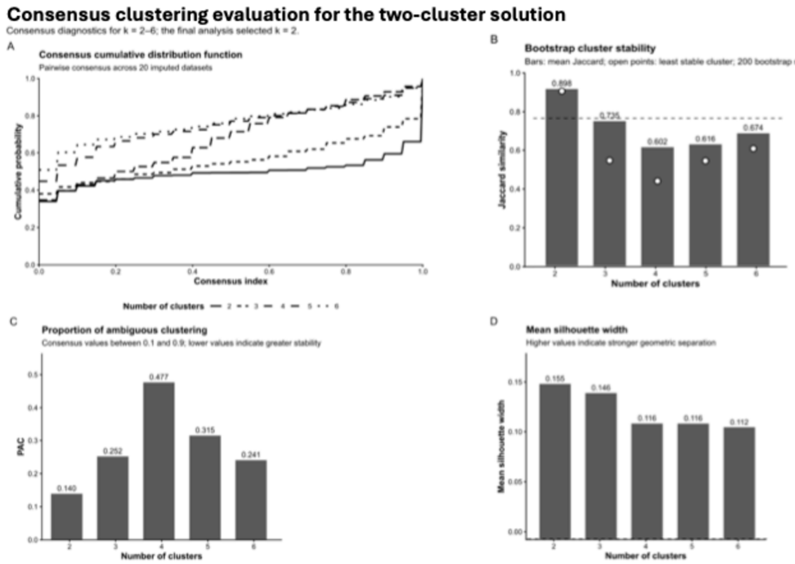

Figure S1. Consensus clustering evaluation for the two-cluster solution. (A) Consensus cumulative distribution functions for solutions with k= 2-6 across 20 multiply imputed datasets. (B) Bootstrap cluster stability expressed as mean Jaccard similarity coefficients; open circles represent the least stable individual cluster, and the dashed horizontal line indicates a Jaccard coefficient of 0.75. (C) PAC, defined as the proportion of pairwise consensus values between 0.10 and 0.90; lower values indicate greater stability. (D) Mean silhouette width, with higher values indicating better geometric separation.

Table S1. Resting echocardiographic characteristics stratified by cluster.

Resting Echocardiography	Cluster 1	Cluster 2	Missing C1	Missing C2	*P* Value	Effect size
Rhythm during echocardiography			5/66	10/87	<0.001	0.35
SR	38 (62.3%)	70 (90.9%)				
AF	20 (32.8%)	6 (7.8%)				
Atrial flutter	2 (3.3%)	1 (1.3%)				
SV runs	1 (1.6%)	0 (0.0%)				
LV end-diastolic diameter (LVEDD) (mm)	48.51 ± 8.16	48.28 ± 8.84	1/66	4/87	0.870	0.03
Interventricular septal thickness (IVSED) (mm)	12.69 ± 4.86	13.90 ± 10.13	1/66	4/87	0.342	-0.15
Posterior wall thickness (PWED) (mm)	11.17 ± 1.86	11.86 ± 8.67	1/66	4/87	0.483	-0.10
LV mass index (LVMI) (g/m^{2})	109.10 ± 26.70	110.48 ± 29.50	1/66	7/87	0.769	-0.05
LV ejection fraction (LVEF) (%)	55.29 ± 6.35	56.89 ± 5.84	0/66	1/87	0.112	-0.26
LA end-systolic volume (LAVES) (ml)	98.55 ± 43.22	73.63 ± 19.29	10/66	13/87	<0.001	0.78
LA volume index, biplane (LAVI) (ml/m^{2})	53.13 ± 23.55	38.82 ± 9.10	2/66	2/87	<0.001	0.84
E wave velocity (cm/s)	102.95 ± 32.15	80.67 ± 22.86	1/66	2/87	<0.001	0.81
A wave velocity in SR only (cm/s)	71.74 ± 34.75	74.28 ± 25.07	9/38	6/70	0.726	-0.09
Lateral e′ velocity (cm/s)	8.02 ± 2.67	7.36 ± 2.16	1/66	3/87	0.106	0.27
Medial e′ velocity (cm/s)	5.97 ± 2.22	6.03 ± 1.71	3/66	4/87	0.858	-0.03
Mean e′ velocity (cm/s)	7.02 ± 2.10	6.69 ± 1.50	3/66	4/87	0.292	0.18
E/e′	16.11 ± 8.40	12.38 ± 3.84	3/66	4/87	0.002	0.60

Resting Echocardiography	Cluster 1	Cluster 2	Missing C1	Missing C2	*P* Value	Effect size
E/e′ during Valsalva	11.33 ± 3.24	9.94 ± 2.64	59/66	75/87	0.354	0.47
LA reservoir strain, 2-chamber (%)	13.35 ± 6.83	19.20 ± 7.86	9/66	22/87	<0.001	-0.79
LA conduit strain, 2-chamber (%)	-9.65 ± 4.84	-10.65 ± 5.08	9/66	22/87	0.270	0.20
LA contraction strain, 2-chamber only in SR-Echo (%)	-5.29 ± 5.82	-9.55 ± 6.48	3/38	11/70	0.002	0.68
LA reservoir strain, 4-chamber (%)	12.62 ± 6.27	20.66 ± 8.46	6/66	13/87	<0.001	-1.06
LA conduit strain, 4-chamber (%)	-9.13 ± 4.09	-11.40 ± 4.89	6/66	13/87	0.004	0.50
LA contraction strain, 4-chamber only in SR-Echo (%)	-5.58 ± 4.45	-10.12 ± 6.72	1/38	3/70	<0.001	0.75
LA reservoir strain, 3-chamber (%)	11.01 ± 4.78	18.08 ± 8.29	14/66	31/87	<0.001	-1.03
LA conduit strain, 3-chamber (%)	-9.15 ± 4.64	-10.54 ± 4.72	14/66	31/87	0.125	0.30
LA contraction strain, 3-chamber only in SR-Echo (%)	-3.50 ± 4.81	-8.58 ± 7.39	4/38	20/70	<0.001	0.78
RA reservoir strain (%)	17.37 ± 10.82	28.30 ± 12.38	21/66	40/87	<0.001	-0.93
RA conduit strain (%)	-11.88 ± 6.46	-16.86 ± 7.98	21/66	40/87	0.001	0.68
RA contraction strain only in SR-Echo (%)	-7.83 ± 8.37	-11.89 ± 8.13	8/38	27/70	0.044	0.49
Baseline TR Vmax (m/s)	2.65 ± 0.80	1.38 ± 1.18	8/66	16/87	<0.001	1.23
TR pressure gradient (mmHg)	30.65 ± 13.73	13.14 ± 12.19	8/66	16/87	<0.001	1.35
sPAP (mmHg)	41.13 ± 16.07	28.75 ± 6.91	27/66	71/87	<0.001	0.87

Resting echocardiographic parameters are shown for patients in Cluster 1 and Cluster 2, including missing data counts and p-values. Transmitral A-wave velocity and LA/RA contraction strain were analysed only in patients in sinus rhythm during resting echocardiographic acquisition; missing values for these variables are therefore reported relative to the sinus-rhythm subgroup (Cluster 1, n = 38; Cluster 2, n = 70). Numeric variables are shown as mean ± SD. Categorical variables are shown as n (%). P-values were calculated using Welch's t-test for continuous variables and the chi-square test or Fisher's exact test for categorical variables. Effect sizes are reported as Hedges' g for Welch comparisons, rank-biserial r for Wilcoxon comparisons, and Cramér's V for categorical comparisons.

Table S2. Stress echocardiographic characteristics stratified by cluster.

Stress Echocardiography	Cluster 1	Cluster 2	Missing C1	Missing C2	*P* Value	Effect size
Rhythm during stress echocardiography			24/66	24/87	0.093	0.21
SR	33 (78.6%)	58 (92.1%)				
AF	8 (19.0%)	5 (7.9%)				
ventricular bigeminy	1 (2.4%)	0 (0.0%)				
Stress echocardiography available	43 (65.2%)	63 (72.4%)	0/66	0/87	0.431	0.08
Stress echocardiography analysed	30 (45.5%)	41 (47.1%)	0/66	0/87	0.967	0.02
Workload at test termination (W)	64.06 ± 21.94	75.00 ± 21.82	34/66	44/87	0.036	-0.49
LA reservoir strain, 4-chamber (%)						
Rest	15.30 ± 5.73	19.04 ± 10.03	37/66	47/87	0.055	-0.43
25 W	17.60 ± 6.17	22.74 ± 8.82	50/66	50/87	0.020	-0.62
50 W	21.10 ± 10.94	24.15 ± 11.04	44/66	59/87	0.335	-0.27
75 W	15.72 ± 5.31	24.74 ± 12.66	53/66	65/87	0.006	-0.83
100 W	8.83 ± 7.85	25.35 ± 10.10	63/66	75/87	0.039	-1.59
Post-Stress	17.27 ± 6.16	22.42 ± 8.88	45/66	55/87	0.016	-0.64
E/e′						
Rest	14.67 ± 6.99	10.57 ± 4.15	36/66	40/87	0.006	0.75

Stress Echocardiography	Cluster 1	Cluster 2	Missing C1	Missing C2	*P* Value	Effect size
25 W	12.91 ± 5.50	11.95 ± 3.95	40/66	41/87	0.442	0.21
50 W	13.92 ± 6.94	12.91 ± 5.89	41/66	46/87	0.548	0.16
75 W	14.82 ± 6.77	12.96 ± 7.45	52/66	56/87	0.415	0.25
100 W	12.68 ± 7.27	13.39 ± 8.69	62/66	71/87	0.873	-0.08
Post-Stress	14.37 ± 7.00	11.79 ± 4.12	37/66	46/87	0.082	0.47
Maximum systolic blood pressure (mmHg)	152.77 ± 28.57	161.36 ± 28.54	35/66	40/87	0.198	-0.30
Maximum diastolic blood pressure (mmHg)	77.58 ± 14.86	85.56 ± 14.61	35/66	42/87	0.024	-0.54
Maximum heart rate (bpm)	94.85 ± 18.34	105.47 ± 20.04	33/66	40/87	0.017	-0.54
Dyspnea	8 (30.8%)	9 (24.3%)	40/66	50/87	0.780	0.07
Cardiac arrhythmias	4 (14.3%)	3 (8.1%)	38/66	50/87	0.453	0.10
Reason for termination			40/66	54/87	0.035	0.45
Dyspnea	6 (23.1%)	7 (21.2%)				
Peripheral fatigue	7 (26.9%)	18 (54.5%)				
Peripheral fatigue and dyspnea	8 (30.8%)	2 (6.1%)				
Leg pain	4 (15.4%)	3 (9.1%)				
Blood pressure-drop, ~~TV insufficiency~~ tricuspid regurgitation under higher stress	1 (3.8%)	0 (0.0%)				
Blood pressure-increase	0 (0.0%)	2 (6.1%)				
~~Doctors'~~ physician-decision and e'-wave fusion	0 (0.0%)	1 (3.0%)				
Termination before 100 W	33 (89.2%)	44 (80.0%)	29/66	32/87	0.378	0.12
Peak stage, LA reservoir strain 2-chamber			49/66	66/87	0.053	0.45
25 W	5 (29.4%)	5 (23.8%)				
50 W	5 (29.4%)	5 (23.8%)				
75 W	5 (29.4%)	1 (4.8%)				
100 W	2 (11.8%)	10 (47.6%)				
Peak workload, LA reservoir strain 2-chamber (W)	55.88 ± 25.81	69.05 ± 32.50	49/66	66/87	0.173	-0.43
Peak LA reservoir strain, 2-chamber (%)	18.82 ± 6.17	23.58 ± 9.18	49/66	66/87	0.065	-0.58
Absolute change in LA reservoir strain, 2-chamber (%)	2.24 ± 4.67	-0.31 ± 6.55	54/66	72/87	0.251	0.43
Relative change in LA reservoir strain, 2-chamber (%)	22.73 ± 39.43	3.48 ± 31.91	54/66	72/87	0.185	0.53
Peak stage, LA reservoir strain 4-chamber			42/66	47/87	0.013	0.40
25W	0 (0.0%)	8 (20.0%)				
50W	11 (45.8%)	9 (22.5%)				
75W	10 (41.7%)	11 (27.5%)				
100W	3 (12.5%)	12 (30.0%)				
Peak workload, LA reservoir strain 4-chamber (W)	66.67 ± 17.55	66.88 ± 27.96	42/66	47/87	0.971	-0.01
Peak LA reservoir strain, 4-chamber (%)	19.29 ± 10.37	23.92 ± 11.40	42/66	47/87	0.102	-0.41
Absolute change in LA reservoir strain, 4-chamber (%)	4.64 ± 9.80	4.80 ± 10.61	42/66	48/87	0.951	-0.02
Relative change in LA reservoir strain, 4-chamber (%)	36.99 ± 67.18	46.60 ± 73.68	42/66	48/87	0.598	-0.13

Stress echocardiographic parameters, including cardiac rhythm during exercise, exercise capacity, exercise-related changes in left atrial function, diastolic function, hemodynamic response, and reasons for exercise termination, are shown for patients in Cluster 1 and Cluster 2, including missing data counts and *p*-values. Numeric variables are shown as mean ± SD. Categorical variables are

shown as n (%). Because measurement availability decreased substantially with increasing workload, particularly at 75 and 100 W, workload-specific comparisons at advanced exercise stages should be considered exploratory. *P*-values were calculated using Welch's t-test for continuous variables and the chi-square test or Fisher's exact test for categorical variables. Effect sizes are reported as Hedges' g for Welch comparisons, rank-biserial r for Wilcoxon comparisons, and Cramér's V for categorical comparisons.

Table S3. Minnesota Living with Heart Failure Questionnaire (MLHFQ) item scores stratified by cluster.

Minnesota Living with Heart Failure Questionnaire (MLHFQ) items	Cluster 1	Cluster 2	Missing C1	Missing C2	*P* Value	BH/FDR q	Effect size
MLHFQ 1: Swelling of ankles, legs, etc.	1.44 ± 1.50	1.15 ± 1.44	14/66	15/87	0.262	0.305	0.11
MLHFQ 2: Need to rest during the day	2.42 ± 1.47	1.70 ± 1.59	14/66	14/87	0.008	0.029	0.27
MLHFQ 3: Difficulty walking or climbing stairs	2.79 ± 1.55	2.18 ± 1.58	14/66	14/87	0.032	0.083	0.22
MLHFQ 4: Difficulty with housework or gardening	2.56 ± 1.67	1.68 ± 1.53	16/66	14/87	0.005	0.020	0.30
MLHFQ 5: Difficulty going out of the house	1.44 ± 1.51	0.92 ± 1.39	14/66	14/87	0.040	0.084	0.20
MLHFQ 6: Difficulty sleeping at night	2.08 ± 1.64	1.55 ± 1.69	14/66	14/87	0.057	0.097	0.20
MLHFQ 7: Difficulty socializing with family or friends	1.41 ± 1.63	0.70 ± 1.28	15/66	14/87	0.003	0.019	0.28
MLHFQ 8: Difficulty working to earn a living	0.06 ± 0.31	0.46 ± 1.18	16/66	16/87	0.026	0.077	-0.13
MLHFQ 9: Difficulty with recreation, sports, or hobbies	2.06 ± 1.65	1.72 ± 1.58	17/66	15/87	0.256	0.305	0.12
MLHFQ 10: Impaired sexual activity	1.87 ± 2.12	1.45 ± 1.79	19/66	18/87	0.361	0.399	0.09
MLHFQ 11: Eating less of the foods you like	0.88 ± 1.37	0.41 ± 0.89	14/66	14/87	0.044	0.084	0.17
MLHFQ 12: Shortness of breath	2.40 ± 1.68	1.38 ± 1.45	16/66	14/87	<0.001	0.017	0.34
MLHFQ 13: Fatigue, tiredness, or low energy	2.57 ± 1.42	1.75 ± 1.43	15/66	14/87	0.002	0.017	0.33
MLHFQ 14: Hospitalization	0.65 ± 1.47	0.25 ± 0.85	15/66	15/87	0.156	0.204	0.09
MLHFQ 15: Cost of medical care	0.78 ± 1.33	0.69 ± 1.34	15/66	15/87	0.789	0.789	0.02
MLHFQ 16: Medication side effects	1.35 ± 1.67	0.88 ± 1.47	15/66	15/87	0.039	0.084	0.19
MLHFQ 17: Feeling like a burden to family	0.80 ± 1.37	0.38 ± 0.89	15/66	14/87	0.060	0.097	0.15
MLHFQ 18: Feeling loss of control over life	0.92 ± 1.59	0.33 ± 0.75	15/66	15/87	0.080	0.120	0.14
MLHFQ 19: Worry related to heart failure	1.65 ± 1.67	0.82 ± 1.25	17/66	16/87	0.004	0.019	0.29
MLHFQ 20: Difficulty remembering things	1.16 ± 1.35	1.16 ± 1.18	16/66	14/87	0.721	0.758	-0.04

Minnesota Living with Heart Failure Questionnaire (MLHFQ) items	Cluster 1	Cluster 2	Missing C1	Missing C2	*P* Value	BH/FDR q	Effect size
MLHFQ 21: Feeling depressed	1.08 ± 1.49	0.60 ± 1.10	15/66	14/87	0.089	0.125	0.16

Individual item scores of the Minnesota Living with Heart Failure Questionnaire (MLHFQ) are shown for patients in Cluster 1 and Cluster 2, including missing data counts and p-values. Numeric variables are shown as mean ± SD. Higher scores indicate greater impairment in health-related quality of life. Individual MLHFQ items were compared between clusters using the Wilcoxon rank-sum test. Effect sizes are reported as rank-biserial correlations, with positive values indicating higher item scores in Cluster 1. For the 21 individual item comparisons, false discovery rate control was performed using the Benjamini-Hochberg procedure. No additional family-wise error rate correction, such as Bonferroni correction, was applied.

Supplementary Table S4: Missingness of the ten variables used for consensus clustering.

Missingness of the 10 clustering variables			
**Clustering variable**	**Overall missing, n/N (%)**	**Cluster 1, n/N (%)**	**Cluster 2, n/N (%)**
Body mass index	0/153 (0.0%)	0/66 (0.0%)	0/87 (0.0%)
LAVI, biplane	4/153 (2.6%)	2/66 (3.0%)	2/87 (2.3%)
Mean E/e′	7/153 (4.6%)	3/66 (4.5%)	4/87 (4.6%)
TR pressure gradient	24/153 (15.7%)	8/66 (12.1%)	16/87 (18.4%)
log(NT-proBNP)	0/153 (0.0%)	0/66 (0.0%)	0/87 (0.0%)
eGFR, CKD-EPI 2021	0/153 (0.0%)	0/66 (0.0%)	0/87 (0.0%)
HbA1c	0/153 (0.0%)	0/66 (0.0%)	0/87 (0.0%)
Hemoglobin	0/153 (0.0%)	0/66 (0.0%)	0/87 (0.0%)
LA reservoir strain, 4-chamber	19/153 (12.4%)	6/66 (9.1%)	13/87 (14.9%)
LV mass index	8/153 (5.2%)	1/66 (1.5%)	7/87 (8.0%)
Overall, 46 of 153 patients (30.1%) had at least one missing value among the ten variables used for clustering.			

Missing values are presented for the overall cohort and separately for Cluster 1 and Cluster 2. Overall, 46 of 153 patients (30.1%) had at least one missing value among the ten clustering variables.

Table S5. Complete-case sensitivity analysis of the clustering solution.

**Complete-case sensitivity analysis**	
**Panel A.** Sample composition and agreement with the primary consensus clustering	
**Measure**	**Result**
Original cohort	153
Complete cases for all 10 clustering variables	107/153 (69.9%)
Patients with ≥1 missing clustering variable	46/153 (30.1%)
Primary Cluster 1 among complete cases	50/107 (46.7%)
Primary Cluster 2 among complete cases	57/107 (53.3%)
Complete-case Cluster 1	59/107 (55.1%)
Complete-case Cluster 2	48/107 (44.9%)
Same cluster assignment	94/107 (87.9%)
Changed cluster assignment	13/107 (12.1%)
Adjusted Rand index	0.569

**Panel B.** Standardized clustering-variable profiles in the complete-case solution			
**Clustering variable**	**Complete-case Cluster 1, mean z**	**Complete-case Cluster 2, mean z**	**Direction in Cluster 1**
BMI	−0.125	+0.154	Slightly lower
Mean E/e′	+0.177	−0.218	Higher
HbA1c	−0.073	+0.090	Slightly lower

Complete-case sensitivity analysis			
**Panel A.** Sample composition and agreement with the primary consensus clustering			
Hemoglobin	−0.233	+0.287	Lower
LAVI	+0.280	−0.344	Higher
LVMI	−0.277	+0.340	Lower
log (NT-proBNP)	+0.525	−0.646	Higher
LA reservoir strain	−0.433	+0.533	Lower
TR pressure gradient	+0.503	−0.618	Higher
eGFR	−0.390	+0.479	Lower

The analysis included 107 patients with complete data for all ten clustering variables. K-means clustering was repeated using the primary preprocessing approach. Cluster labels were aligned for comparison, and agreement was assessed using the adjusted Rand index. Panel B shows mean z-scores.

Table S6. Sinus-rhythm sensitivity analysis.

Sinus-rhythm sensitivity analysis			
**Panel A.** Rhythm status and composition of the sensitivity-analysis cohort			
**Variable**	**Overall cohort**	**Cluster 1**	**Cluster 2**
Total, n	153	66	87
Sinus rhythm during resting echocardiography, n (%)	108 (70.6%)	38 (57.6%)	70 (80.5%)
Non-sinus rhythm during resting echocardiography, n (%)	30 (19.6%)	23 (34.8%)	7 (8.0%)
Missing rhythm information, n (%)	15 (9.8%)	5 (7.6%)	10 (11.5%)
History of atrial fibrillation among patients in sinus rhythm, n (%)	55/108 (50.9%)	22/38 (57.9%)	33/70 (47.1%)
**Panel B.** Atrial measures in patients in sinus rhythm during resting echocardiography			
**Variable**	**Cluster 1**	**Cluster 2**	**p-value**
LA reservoir strain (LASr), %	15.11 ± 6.09, n = 37	21.56 ± 8.30, n = 67	<0.001
LAVI, mL/m²	48.07 ± 16.66, n = 38	38.62 ± 8.51, n = 68	0.002
LA stiffness index	1.37 ± 1.07, n = 34	0.68 ± 0.38, n = 64	<0.001
LA contraction strain (LASct), %	-5.58 ± 4.45, n = 37	-10.12 ± 6.72, n = 67	<0.001
**Panel C.** External clinical measures in the sinus-rhythm subgroup			
**Variable**	**Cluster 1**	**Cluster 2**	**p-value**
Peak VO_2, L/min	1.03 ± 0.32, n = 36	1.27 ± 0.42, n = 64	0.002
VE/VCO_2 slope	40.48 ± 10.86, n = 36	35.29 ± 7.78, n = 64	0.014
6-minute walk distance, m	354.27 ± 110.35, n = 37	419.14 ± 118.13, n = 70	0.006
MLHFQ total score	34.06 ± 19.26, n = 33	23.86 ± 17.15, n = 58	0.014

The analysis was restricted to patients in sinus rhythm during resting echocardiography. Continuous variables are presented as mean ± SD and were compared using Welch’s t-test; categorical variables are presented as n (%).

Table S7. LVEF ≥ 50% sensitivity.

Sensitivity analysis in patients with LVEF ≥50%	
**Panel A.** Robustness of cluster assignment	
**Measure**	**Result**
Patients included	138
Patients excluded (LVEF < 50%)	14

Sensitivity analysis in patients with LVEF ≥50%	
Patients excluded due to missing LVEF in Echocardiography	1
Cluster 1 LVEF ≥50%	56
Cluster 2 LVEF ≥50%	82
Adjusted Rand index vs. primary clustering	0.914
Preserved original cluster assignment	135/138 (97.8%)
Changed cluster assignment	3/138 (2.2%)
Main Cluster 1 → Cluster 2 LVEF ≥50%	3
Main Cluster 2 → Cluster 1 LVEF ≥50%	0

Panel B. Functional and patient-reported outcomes

**Outcome**	**Cluster 1**	**Cluster 2**	**Missing C1**	**Missing C2**	**p-value**
Peak VO_2, L/min	1.11 ± 0.42	1.33 ± 0.42	5/56	6/82	0.004
VE/VCO_2 slope	40.06 ± 10.34	34.28 ± 6.37	5/56	7/82	<0.001
6-minute walk distance, m	366.66 ± 110.88	437.98 ± 108.89	3/56	1/82	<0.001
MLHFQ total score	31.30 ± 17.56	20.88 ± 16.75	10/56	13/82	0.002

Panel C. Clinical outcomes

**Outcome**	**Cluster 1**	**Cluster 2**	**Missing C1**	**Missing C2**	**Comparison**	**p-value**
≥1 all-cause hospitalization	12/44 (27.3%)	10/57 (17.5%)	12/56	25/82	OR 1.75 (0.61-5.13)	0.331
≥2 general practitioner visits	37/43 (86.0%)	45/56 (80.4%)	13/56	26/82	OR 1.50 (0.46-5.44)	0.593
≥2 cardiologist visits	24/43 (55.8%)	22/57 (38.6%)	13/56	25/82	OR 2.00 (0.83-4.86)	0.107
General practitioner visits, n	6.41 ± 6.16	4.90 ± 4.60	13/56	26/82		0.184
Cardiologist visits, n	1.73 ± 1.79	1.55 ± 2.30	13/56	25/82		0.661
All-cause hospitalizations, n	0.43 ± 0.82	0.25 ± 0.61	12/56	25/82		0.210
Total days hospitalized, d	3.53 ± 5.91	2.41 ± 4.79	13/56	26/82		0.312
NYHA improvement	4/45 (8.9%)	15/57 (26.3%)	11/56	25/82		
NYHA unchanged	35/45 (77.8%)	35/57 (61.4%)				0.081†
NYHA worsening	6/45 (13.3%)	7/57 (12.3%)				

†Fisher's exact test comparing improved, unchanged, and worsened NYHA categories. Continuous variables are presented as mean ± SD and compared using Welch's t-test. Binary outcomes are presented as n/N (%) and were compared using Fisher's exact test; odds ratios shown in Panel C are estimates for Cluster 1 versus Cluster 2. NYHA change was analyzed in patients with assessments available at both baseline and follow-up. P-values are nominal and should be interpreted as exploratory.
